# Role of WDR66 in Stemness, Therapy Resistance and Tumor microenvironment modulation in Head and Neck Cancer

**DOI:** 10.7150/ijbs.130010

**Published:** 2026-05-22

**Authors:** Laura Sanchez-Diaz, Asuncion Espinosa-Sánchez, Amancio Carnero

**Affiliations:** 1 Instituto de Biomedicina de Sevilla (IBIS)/HUVR/CSIC/Universidad de Sevilla, Sevilla, Spain.; 2 CIBER de Cancer (CIBERONC), Instituto de Salud Carlos III, Madrid, Spain.

**Keywords:** WDR66, stemness, therapy resistance, immune modulation, head and neck cancer

## Abstract

Head and neck cancer comprises a variety of malignant tumors affecting regions such as the oral cavity, pharynx, larynx, paranasal sinuses, nasal cavity and salivary glands. These tumors are highly heterogeneous, making it difficult to identify specific biomarkers and effective treatments, increasing mortality and recurrence rates. Databases analyzed to identify genes with altered expression in tumor tissue showed WDR66 able predict prognosis and survival, in HNSCC, but whose involvement in tumorigenesis is unknown. High WDR66 expression was associated with a worse prognosis, although its overexpression did not significantly alter basic tumor properties in cancer cells *in vitro* or *in vivo*. However, we observed an increase in pluripotency factors (OCT3/4, SOX2, KLF4, LIN28), resistance to chemotherapies and a decrease in apoptosis. Consistently, decreasing WDR66 expression using CRISPR showed a reduction in tumorsphere formation, lower expression of pluripotency-related genes, decreased cell migration, and increased sensitivity to cisplatin. These effects were more pronounced *in vivo*, suggesting a relevant role of WDR66 in the tumor microenvironment. The medium conditioned by cells overexpressing WDR66 promoted migration, maintained the stem cell phenotype, and inhibited immune differentiation, harboring soluble factors involved in progression and metastasis. In this media, we identified several cytokines and growth factors related to inflammation and immune system modulation. In conclusion, WDR66 emerges as a potential biomarker of poor prognosis and a possible predictor of response to immunotherapy, given its impact on cellular pluripotency, treatment resistance and modification of the tumor microenvironment in head and neck cancer.

## Introduction

Head and neck cancer (HNC) is the seventh most prevalent cancer worldwide, with approximately 890,000 new cases and 450,000 deaths annually 1-4. It affects men two to four times more often than women, and its incidence continues to rise. This group of tumors is heterogeneous, both phenotypically and biologically, originating in diverse anatomical sites such as the oral cavity, pharynx, larynx, paranasal sinuses, nasal cavity and salivary glands. The most common subtype (90%) is head and neck squamous cell carcinoma (HNSCC) 3 5.

The main risk factors are alcohol and tobacco use, as well as human papillomavirus (HPV) infection, which is implicated in 30-60% of oropharyngeal carcinomas 5. HPV, especially type 16, is an important prognostic marker, as HPV-positive tumors tend to respond better to therapy and have a longer survival rate 5. In contrast, HPV-negative tumors present a greater number of genetic alterations, such as TP53 mutations, CDKN2A deletions, and CCND1 and PIK3CA amplifications 5-7.

Regarding treatment, combinations of surgery, radiotherapy and chemotherapy are used depending on the stage and location 8. In advanced cases, platinum-based chemotherapy is used, often with cetuximab, which blocks the EGFR receptor. Immunotherapies such as pembrolizumab and nivolumab, PD-1 inhibitors, have also been approved, although they only benefit a limited percentage of patients 8-13. Despite these advances, the long-term survival rate is less than 50%, and in advanced cases it drops to 19% 14, 15.

The cancer stem cell (CSC) theory offers a more precise explanation for tumor progression and therapeutic resistance 15. These cells possess self-renewal capacity and can originate tumor mass, contribute to metastasis, and cause relapse 14, 15. In HNSCC, markers such as CD44, CD133, CD10, CD184 16 or ALDH1, and transcription factors such as SOX2, NANOG, and BMI1 have been proposed to identify CSCs, although their usefulness remains under debate due to tumor heterogeneity 14, 15, 17.

The tumor microenvironment also plays an essential role 15, 18, 19. It is composed of tumor cells, stem cells, immune cells, fibroblasts and components of the extracellular matrix. The interaction between these cells, through signaling and cytokine secretion, regulates progression, angiogenesis, therapeutic resistance and immune evasion 20, 21. Factors such as hypoxia, altered metabolism, angiogenesis and senescence contribute to an environment favorable for tumor progression. Furthermore, exosomes, ncRNA and circulating DNA participate in cell-cell communication, promoting metastasis and treatment resistance 20-22.

Therefore, there is an urgent need to identify new diagnostic and therapeutic biomarkers, as well as specific molecular targets, that will allow for the development of more personalized and effective treatments for this complex and heterogeneous type of cancer.

Although the available information is still limited, WDR66 (WD repeat protein 66), also known as CFAP251 (cilia and flagellum associated protein 251), has begun to be linked to various types of cancer, raising the question of its possible oncogenic role 23-28. Overexpression of WDR66 has been observed in various tumors including oral cavity 29 and esophageal squamous cell carcinoma (ESCC), where significantly elevated expression was found in 96% of cases, correlating with a worse prognosis 23. It has been proposed that it regulates epithelial-mesenchymal transition (EMT), a key process in tumor progression, as its reduction decreases proliferation, migration, and EMT markers 23. In salivary adenoid cystic carcinoma, WDR66 expression is inversely associated with that of PTEN, a tumor suppressor 27. The lncRNA, AK096174, has been identified in gastric cancer that positively regulates WDR66 expression 25. Both genes are located close together in the genome, and AK096174 acts at the post-transcriptional level, promoting malignant characteristics such as cell proliferation and invasion 25. In breast cancer, the lncRNA FBXL19-AS1, stabilized by the protein LIN28A, regulates WDR66 26. This signaling pathway enhances cell migration and invasion and promotes EMT. Blocking any of these components reduces WDR66 expression and inhibits tumor processes 26. In hepatocellular carcinoma (HCC), an interaction between WDR66 and the RNA-binding factor eIF4AIII, which stabilizes its messenger RNA, has been described. Its elevated expression promotes proliferation, migration, and EMT 30. The microRNA miR-2113 has also been shown to negatively regulate WDR66, suggesting a model of competitive regulation between eIF4AIII and miR-2113 on WDR66 30. Based on these observations, it is postulated that WDR66 could become a useful biomarker in some cancer types, as well as a potential therapeutic target, especially in head and neck tumors, where its role has not yet been extensively characterized.

In all these tumor contexts, WDR66 appears overexpressed and is associated with a worse prognosis, although the exact mechanisms of its oncogenic action are not yet fully understood. Although available studies are still limited, current findings suggest that WDR66 could play an oncogenic role, being regulated by lncRNAs and RBPs in multiple cancer types. In this regard, further research into their mechanisms of action is a priority, particularly in head and neck tumors, where there is an urgent need to identify new biomarkers and effective therapeutic targets to improve patient prognosis and treatment.

## Results

### Analysis of the role of WDR66 in head and neck cancer databases

**Figure 1:**
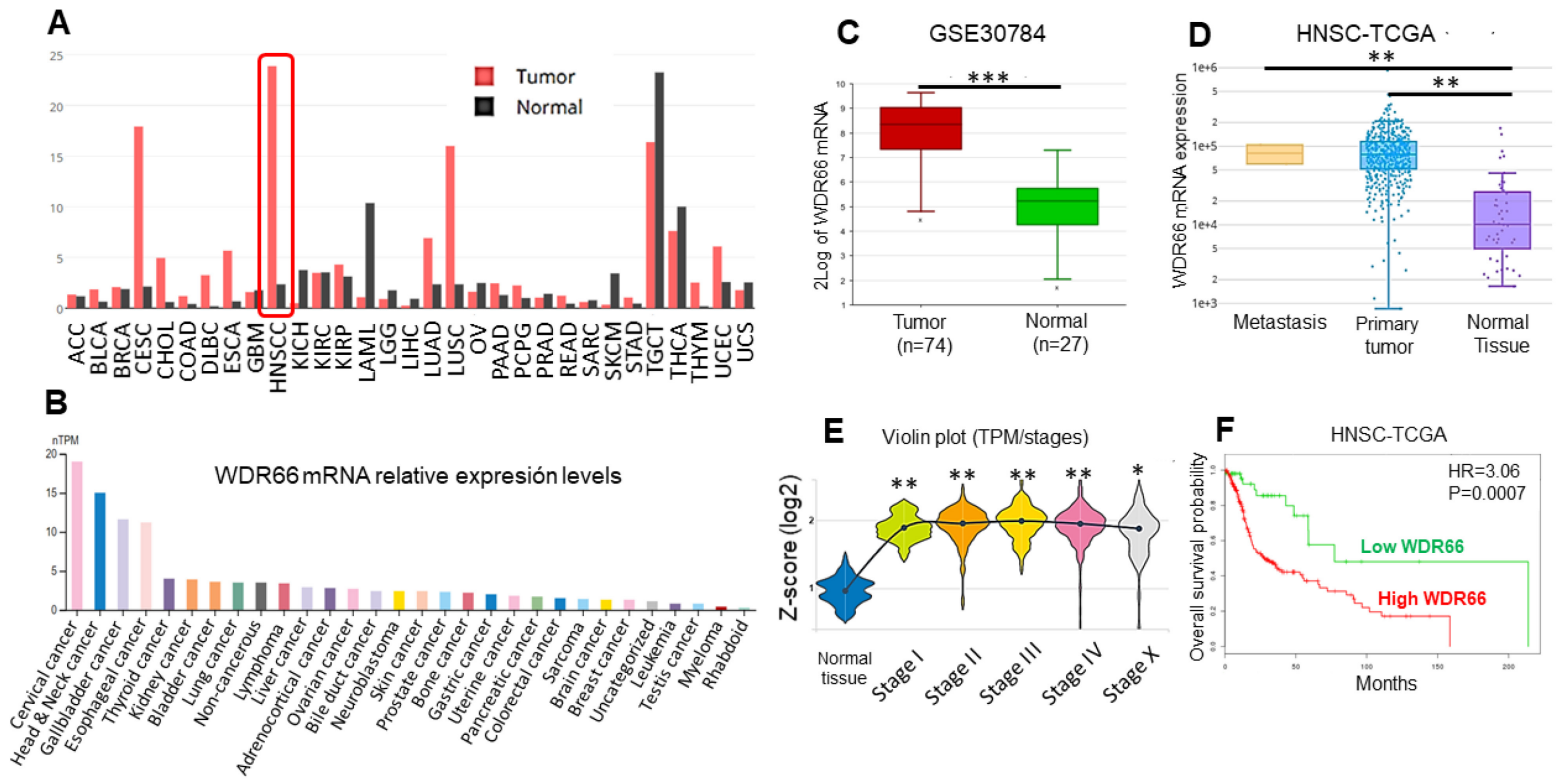
**Study of the role of WDR66 in patient databases. (A)** WDR66 expression levels in different tumors in the TCGA database (canSAR). **(B)** WDR66 expression levels in human tumor cell lines (The Human Protein Atlas). **(C)** WDR66 expression levels in tumor versus normal tissue in the GSE30784 database of oral cavity tumors (R2) analyzed using a moderated t-test (limma) with Benjamini-Hochberg correction. **(D)** WDR66 expression levels in metastases, tumor and normal tissue in the HNSC-TCGA database (DriverDBV4), analyzed using the Wilcoxon rank-sum test. **(E)** WDR66 expression levels in normal tissue and different tumor stages in the HNSC-TCGA database (canSAR), analyzed using Kruskal-Wallis with Dunn-type post hoc test. **(F)** Kaplan-Meier curve based on overall survival according to WDR66 expression in the HNSC-TCGA database (SurvExpress), analyzed using the log-rank (Mantel-Cox) test. High and low WDR66 groups were defined by SurvExpress using its optimized risk stratification based on the Cox proportional hazards model. *p<0.05, **p<0.01, ***p<0.001.

WDR66 gene expression was analyzed in different cancer types using several databases. It was observed that WDR66 is most highly expressed in head and neck cancer (TCGA, via canSAR), followed by testicular and cervical cancer (**Fig. 1A**). In human tumor cell lines (The Human Protein Atlas data), high levels of expression were detected in cervical and head and neck cancer (**Fig. 1B**). When specifically studying head and neck cancer, analysis of the GSE30784 dataset (GEO) of oral cavity tumors showed greater WDR66 expression was found in tumor tissue compared to normal tissue (**Fig. 1C**). This overexpression was observed in both primary tumors and metastases (**Fig. 1D**), and remains elevated across different stages of cancer (**Fig. 1E**) in HNSCC-TCGA dataset (DriverDBv4/canSAR). This may occur through DNA demethylation, since tumors show high levels of DNA demethylation (**Supplementary Fig. 1**). Furthermore, high levels of WDR66 are associated with a worse prognosis, as they are associated with lower overall patient survival in HNSCC-TCGA dataset (SurvExpress) (**Fig. 1F**). WDR66 could play a relevant role in head and neck cancer and serve as a potential prognostic biomarker for this disease.

### Effect of WDR66 levels in the tumorigenic properties of HNSCC cell lines

**Figure 2:**
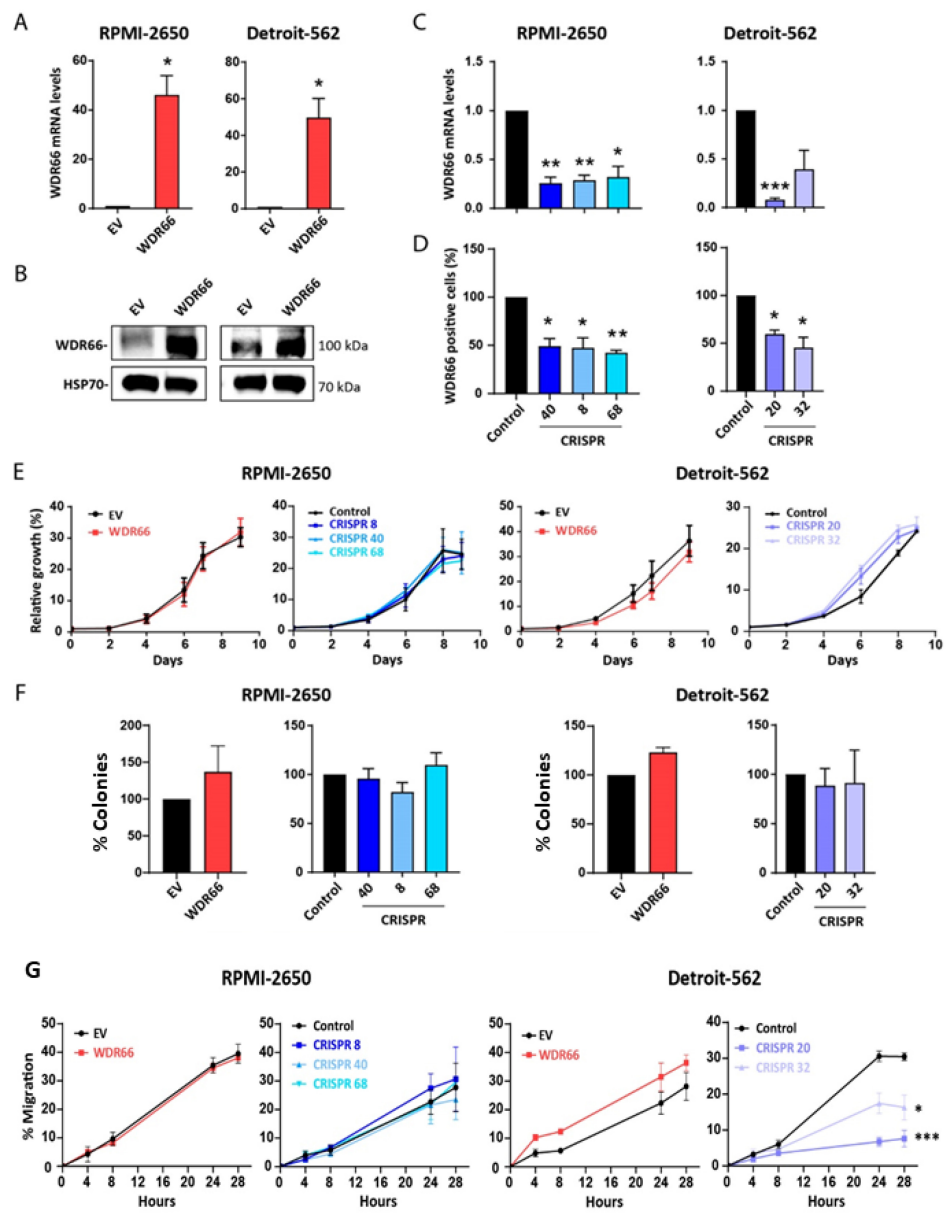
** Effect of WDR66 up/downregulation on proliferative and colony-forming capacity in head and neck cancer cell lines**. **(A)** Validation of WDR66 upregulation in head and neck cancer cells by RT‒qPCR analysis. EV=Empty vector; WDR66= WDR66 overexpressing cells. **(B)** Validation of WDR66 upregulation in head and neck cancer cells by Western Blot analysis. **(C)** Validation of WDR66 downregulation in head and neck cancer cells by RT‒qPCR analysis. **(D)** Validation of WDR66 downregulation in head and neck cancer cells by flow cytometry analysis. **(E)** Growth curve analysis and **(F)** colony formation ability of head and neck cancer cells with WDR66 up/downregulation. **(G)** Wound healing assay in cells up/dowregulating WDR66 relative to control cells. The percentage of wound closure over time is represented. The mean of 3 independent experiments ± SEM is presented. Statistical analysis was performed with Student's t test (*p<0.05; **p<0.01; ***p<0.001). The absence of an asterisk means that the data are not statistically significant.

The role of WDR66 in head and neck cancer was studied by overexpressing this gene in two tumor cell lines of different tissue origin: RPMI-2650 (nose) and Detroit-562 (pharynx). The cells were stably transfected with a plasmid that induced WDR66 overexpression, and its expression was confirmed at both the mRNA and protein levels (**Fig. 2A-B**). CRISPR-Cas9 systems were used to reduce WDR66 gene expression in the same HNSCC cell lines. WDR66 downregulation was validated by RT-qPCR, Western blot, immunofluorescence and flow cytometry, confirming decreased expression of the gene and its protein in the selected clones (**Fig. 2C-D**) (**Supplementary Figures 2 and 3**). Due to differences in CRISPR editing efficiency and clonal viability between the two cell lines, three validated clones were obtained for one model, while only two clones successfully met the editing and quality criteria in the second. The selected clones exhibited consistent phenotypes, ensuring the robustness of the conclusions.

In the overexpression assays, empty vector-transfected cells were used as controls to account for vector related effects. In CRISPR knockout experiments, non-transfected wild type cells were used instead, as CRISPR vectors without guide RNA are not neutral controls due to potential Cas9 associated DNA damage responses.

The functional effects of these WDR66 mRNA and protein level variations were subsequently evaluated. No significant changes in proliferation or clonability were observed in any of the cell lines studied (**Fig. 2E-F**), indicating that WDR66 does not directly influence these basic tumor properties.

Next, we measured the impact of WDR66 on cell migration. In the Detroit-562 cell line, WDR66 reduction by CRISPR significantly decreased migration, whereas WDR66 overexpression showed only a trend toward increased migratory capacity (p = 0.2172) (**Fig. 2G**). These results indicate that the effect of WDR66 on migration is context-dependent, with significant reduction observed in CRISPR clones and a non-significant tendency in overexpressing cells.

### Effect of WDR66 levels in stemness and pluripotency of HNSCC cells

Since no marked phenotypic effects were observed in the tumor lines initially by altering WDR66 levels, we explored the role of WDR66 in the key processes related to cellular pluripotency, EMT and cell death in the context of head and neck cancer.

**Figure 3:**
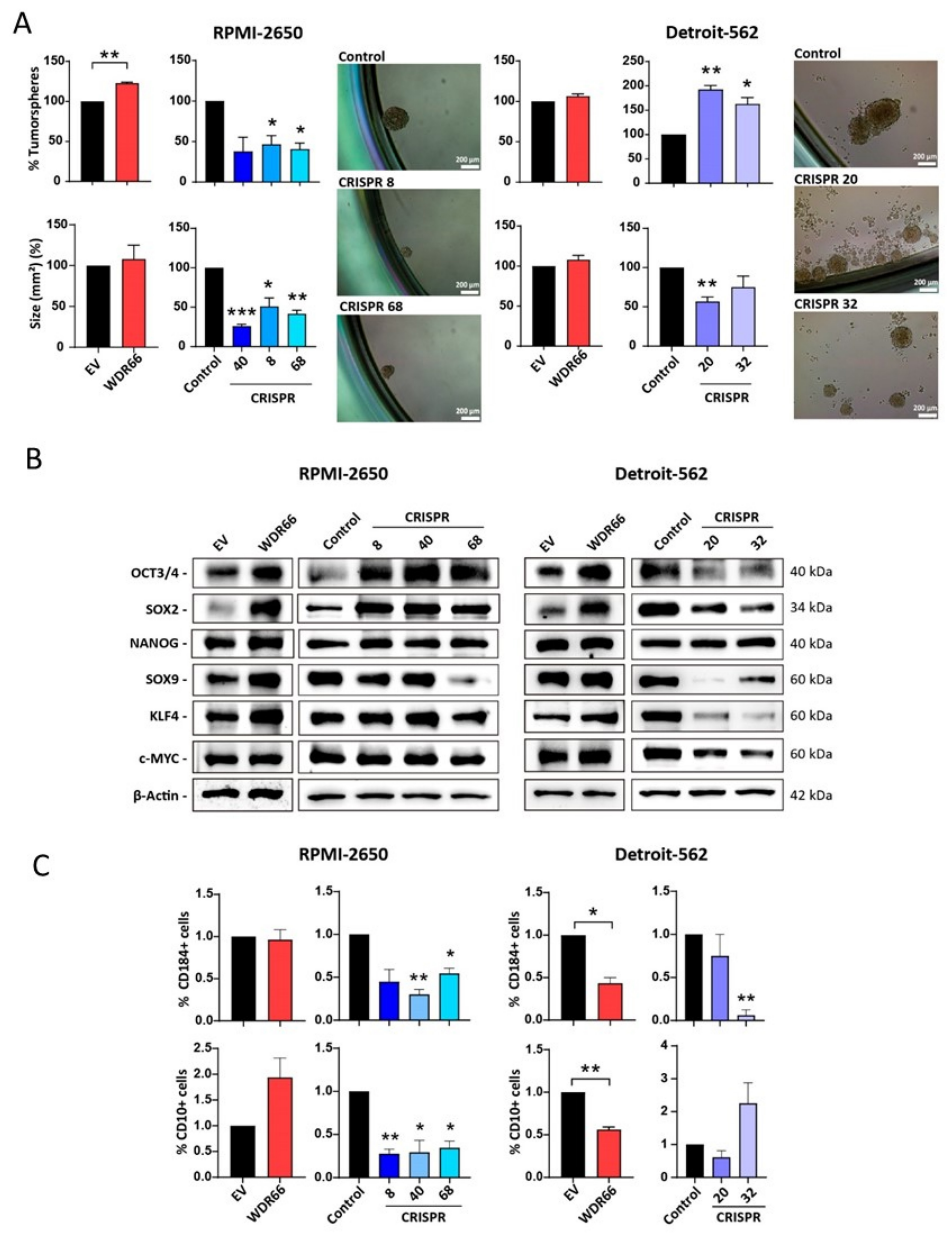
** Effect of WDR66 up/downregulation on stem cell phenotype in head and neck cancer cell lines**. **(A)** Tumorsphere formation assay in head and neck cancer cells with up/downregulated WDR66 and control cells. The result is expressed in % tumorspheres because the control has been normalized to 100%. Below is the area in mm^2^ of the formed tumorspheres. Representative images of the tumorspheres formed in the two cell lines. **(B)** Study of the levels of proteins related to the stem cell phenotype by Western blot in cells with up/downregulation of WDR66 and control cells. **(C)** Quantification by flow cytometry (FACS) of the expression of the surface markers CD184 and CD10 in the up/dowregulation models. The mean of 3 independent experiments ± SEM is presented. Statistical analysis was performed with Student's t test (*p<0.05; **p<0.01; ***p<0.001). The absence of an asterisk means that the data are not statistically significant. EV=Empty vector; WDR66= WDR66 overexpressing cells.

Although an increase in tumorsphere formation was observed in the RPMI-2650 cell line overexpressing WDR66, no clear differences were found in Detroit-562 (**Fig. 3A**). The WDR66 knockdown in RPMI 2650 significantly reduced both the number and size of tumorspheres (**Fig. 3A**). Moreover, CRISPR 20 in Detroit 562 showed a marked reduction in tumorsphere size, whereas the number of tumorspheres showed a slight increase (**Fig. 3A**). These results indicate that the impact of WDR66 on tumorsphere formation is cell line dependent, affecting sphere initiating capacity and growth to different extents in each model. The expression of pluripotency-related transcription factors (such as OCT4, SOX2, NANOG, KLF4, SOX9) was analyzed and an increase in several of them was found, especially in OCT4 and SOX2 in WDR66 overexpressing cell lines (**Fig. 3B**). However, this reduction of stemness genes did not consistently reflect WDR66 downregulation in either cell line (**Fig. 3B**). This suggest that the stemness factors analyzed may not be reliable markers of the stemness phenotype in these cell lines. Additionally, the presence of stem cell-associated surface markers (CD10, CD184) was measured. We found that overexpression of WDR66 showed a trend toward an increase of CD10+ cells in RPMI 2650 (p=0.1313), while a decrease in CD10+ (and CD184+) cells was observed in Detroit 562 (**Fig. 3C**). However, downregulation of WDR66 showed a clear reduction of both CD10+ and CD184+ cells, although in Detroit 562, this reduction showed some variability according to the clone analyzed (**Fig. 3C**).

### Rescue of WDR66 expression restores stemness-associated features in CRISPR-edited HNSCC cells

To further evaluate whether the phenotypic alterations observed upon WDR66 downregulation could be reversed, we performed rescue experiments by restoring WDR66 expression in CRISPR clones from both cell lines, focusing on stemness-related features where the most consistent effects were observed. Efficient restoration of WDR66 expression was confirmed at both mRNA and protein levels by RT-qPCR and Western blot analyses, respectively (**Supplementary Fig. 4A-B**), except in the D20 clone, where a slight increase in WDR66 mRNA levels was observed.

Functionally, rescue of WDR66 expression in tumorsphere formation assays resulted in an increase in tumorsphere number in all CRISPR clones, reaching statistical significance in most cases, while R40 showed a clear increasing trend (p= 0.4583). Regarding tumorsphere size, an increase was also observed in both cell lines compared to their respective CRISPR clones, indicating a recovery of the stemness phenotype (**Supplementary Fig. 4C**).

Altogether, these results support the notion that restoration of WDR66 expression rescues stemness-associated properties in CRISPR-edited cells, reinforcing its role in the regulation of tumor cell plasticity in head and neck cancer.

**Figure 4:**
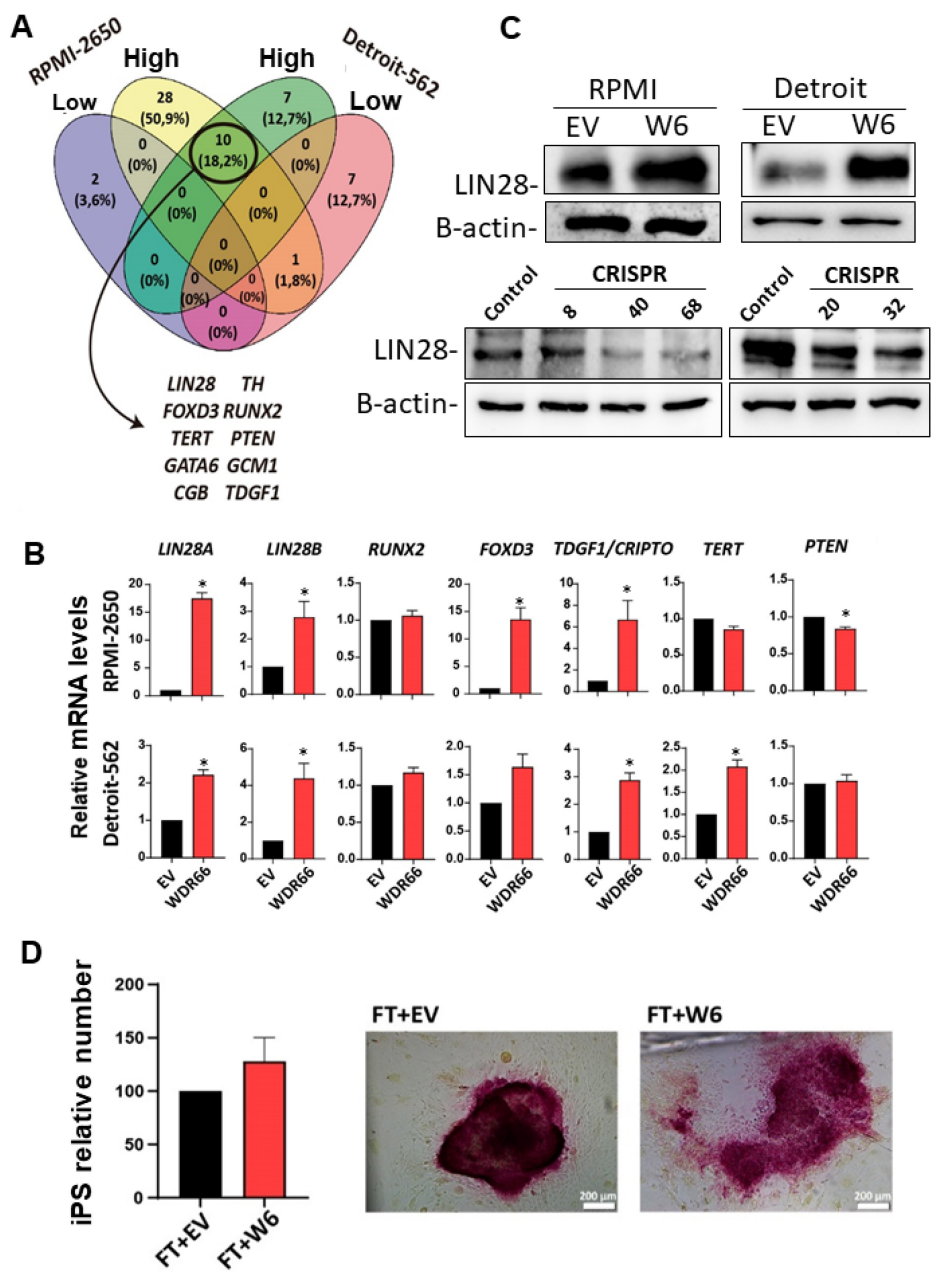
** Study of pluripotency gene expression in WDR66-overexpressing cells. (A)** Venn diagram resulting from the PCR-array of the expression of pluripotency-related genes in WDR66-overexpressing cells and control cells of the RPMI-2650 and Detroit-562 lines. **(B)** Quantification of the mRNA levels of several of the genes obtained in the PCR-array by RT-qPCR in WDR66-overexpressing cells compared to control cells in the three cell lines. **(C)** LIN28A/B protein levels by Western blot in WDR66-overexpressing cells, WDR66 CRISPR and control cells in the two cell lines. **(D)** Effect of WDR66 overexpression on the formation of induced pluripotent stem cells. Left graph, relative number of induced pluripotent fibroblast colonies over time under EV conditions and with the WDR66 plasmid. Right pictures, Representative images of colonies positive for alkaline phosphatase activity. The assays represent the mean of three independent experiments performed in triplicate ± standard error. Statistical analysis was performed using the Student's t-test: *p<0.05, **p<0.01, ***p<0.001; if no asterisk is shown, the statistic was not significant. EV=Empty vector; WDR66= WDR66 overexpressing cells.

To identify these possible regulating stemness factors, a “stemness” PCR array was performed to analyze the expression of a broader set of pluripotency-related genes in the RPMI-2650 and Detroit-562 lines. Ten genes were identified as commonly upregulated in both WDR66-overexpressing lines, including FOXD3, PTEN, TERT, TDGF1, RUNX2 and LIN28 (**Fig. 4A**). The upregulation of some of these genes was validated by RT-qPCR and Western blot, with LIN28A/B being the most notable (**Fig. 4B-C**). Consistently, LIN28 protein levels were reduced in WDR66 CRISPR-edited cells (**Fig. 4C**), supporting a positive regulation of LIN28 by WDR66. Given that LIN28 inhibits microRNAs of the let-7 family, which act as tumor suppressors, these results suggest that WDR66 could be involved in the regulation of the stem cell phenotype and in mechanisms associated with poor prognosis in head and neck cancer, possibly through a direct or indirect interaction with LIN28.

Therefore, we decided to study the possible effect of WDR66 in primary non-tumor cells, to see if it might have any effect on cellular reprogramming. Somatic cells can be reprogrammed into pluripotent cells by expressing the transcription factors OCT3/4, SOX2, KLF4, and c-MYC, used by Shinya Yamanaka 31-34. In our study, we used mouse embryonic fibroblasts (MEFs) infected with expression plasmids for these four transcription factors and with an expression plasmid for a Nanog reporter gene (Nanog-GFP). MEFs were also infected with the WDR66 overexpression plasmid and an empty plasmid (EV) as a control. These infected fibroblasts were seeded on a layer of SNL cells, which acted as a feeder layer and had been inactivated with mitomycin-C. One week after observing the first colonies, an alkaline phosphatase assay was performed to determine if the observed colonies presented phosphatase activity, a typical marker of induced pluripotent stem cells, and it was observed that all of them expressed this marker. We observed that the number of induced pluripotent fibroblast colonies increased slightly when infected with the WDR66 plasmid (**Fig. 4D**). Therefore, these results suggest that, although the differences were not statistically significant, WDR66 could play a role in cellular reprogramming because we observed an increasing trend in the number of induced pluripotent colonies when we overexpressed WDR66.

Subsequently, the impact of WDR66 on epithelial-mesenchymal transition (EMT) was analyzed, revealing increased expression of some EMT-associated genes (such as FOXC2, TWIST1, SNAI1, VIM, CDH2, and β-catenin) that varied depending on the cell line. In RPMI-2650 and Detroit-562, an increase in certain mesenchymal markers was detected, and PCR-array identified the genes SPP1 and SNAI3 as commonly overexpressed, as well as PDGFRB downregulated in both lines** (Supplementary Figure 5)**.

### Effect of WDR66 overexpression on resistance to conventional therapies in head and neck cancer cell lines

**Figure 5:**
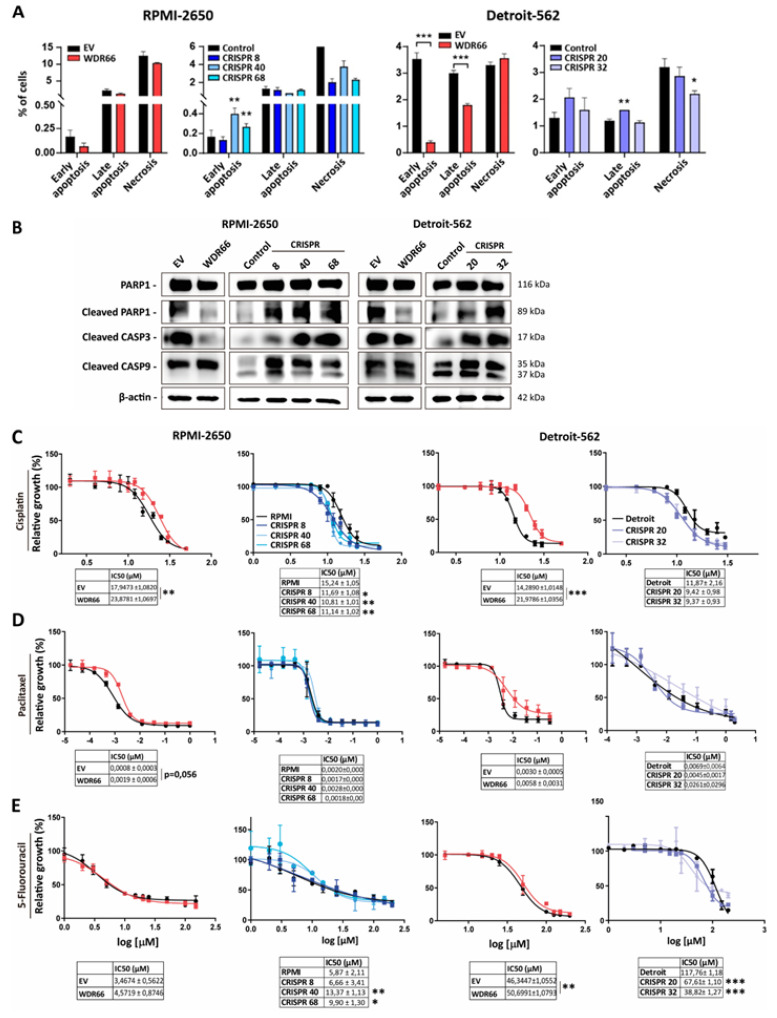
** Effect of WDR66 up/dowregulation on cell death in head and neck cancer cell lines**. **(A)** Quantification of the percentage of cells in early, late apoptosis and necrosis by flow cytometry in head and neck cancer cells with up/dowregulated WDR66, after labeling with Annexin V and propidium iodide. **(B)** Study of the protein levels of Apoptosis-related proteins in WDR66 up/downregulation models by Western blot. The mean of 3 independent experiments ± SEM is presented. Statistical analysis was performed with Student's t test (*p<0.05; **p<0.01; ***p<0.001). The absence of an asterisk means that the data are not statistically significant. **C, D, E)** Effect of WDR66 up/downregulation on resistance to conventional therapies in head and neck cancer cell lines. Representative curves of cell growth when treated with increasing doses of cisplatin **(C)**, paclitaxel **(D)** and 5-fluorouracil **(E)** in head and neck cancer cells with up/downregulated WDR66 and control cells. The mean of 3 independent experiments±SEM is presented. Statistical analysis was performed with Student's t test (*p<0.05; **p<0.01; ***p<0.001).

One possibility for the prevalence of WDR66 upon clinically relevant tumors is the possible involvement in resistance to therapy or to basal cell death, therefore increasing the likelihood of survival of high expressing cells among the tumors. Therefore, the effect of WDR66 on cell death was investigated. Flow cytometry, using Annexin-V and propidium iodide staining, showed a decrease in the proportion of cells undergoing basal apoptosis and necrosis in RPMI-2650 and Detroit-562 cell lines overexpressing WDR66, with a greater incidence in Detroit-562 (**Fig. 5A**). Furthermore, protein studies revealed lower amounts of cleaved PARP1 and caspase-3 under these conditions, suggesting a reduction in apoptotic activation (**Fig. 5B**). On the contrary, when WDR66 was downregulated by CRISPR, we observed increased expression of PARP1, cleaved Caspase 3, and Caspase 9 in both the RPMI-2650 and Detroit-562 CRISPR clones compared to the parental line **(Fig. 5 A-B)**. Together, these findings indicate that WDR66 may play a protective role against cell death in head and neck tumor cells, in addition to participating in cell reprogramming and migration processes.

Next, we examined whether WDR66 levels influence resistance to cell death by conventional therapies in head and neck cancer. Given that WDR66 appears to protect against cell death and is associated with tumor stem cell (CSC) characteristics, its effect against the chemotherapeutics cisplatin, paclitaxel and 5-fluorouracil was evaluated. In *in vitro* cytotoxicity assays, cells overexpressing WDR66 showed a significant increase in resistance to cisplatin, while paclitaxel displayed a trend toward increased resistance in RPMI-2650 cells (p = 0.056) (**Fig. 5C-D**). In the case of 5-fluorouracil, only the Detroit-562 cell line showed a significant increase in resistance (**Fig. 5E**). Next, we set out to study whether the decreased expression of WDR66 played a role in the sensitivity to therapies *in vitro*, since we have observed that cells with WDR66 reduction are more sensitive to cell death. In this case, we observed an increase in the sensitivity to cisplatin of cells with WDR66 reduction in both cell lines compared to the parental cells (**Fig. 5C-E**). When we treated the cells with paclitaxel, we observed no changes in the sensitivity of the CRISPR cells compared to the parental cells. Finally, sensitivity to 5-fluorouracil appears to be increased in CRISPR clones from Detroit-562, but not RPMI-2650 (**Fig. 5C-E**).

These results suggest that WDR66 may contributes to resistance to certain chemotherapeutic treatments, possibly through its association with the tumor stem cell phenotype and its ability to reduce apoptosis.

### Effect on WDR66 levels *in vivo*

WDR66 levels do not affect proliferation or clonability, but it does clearly impact on the characteristics associated with the tumor stem cell phenotype, apoptosis and, on Detroit 562 cell line, in cell migration. This reinforces the potential role of WDR66 as a regulator of tumor aggressiveness in head and neck cancer.

**Figure 6:**
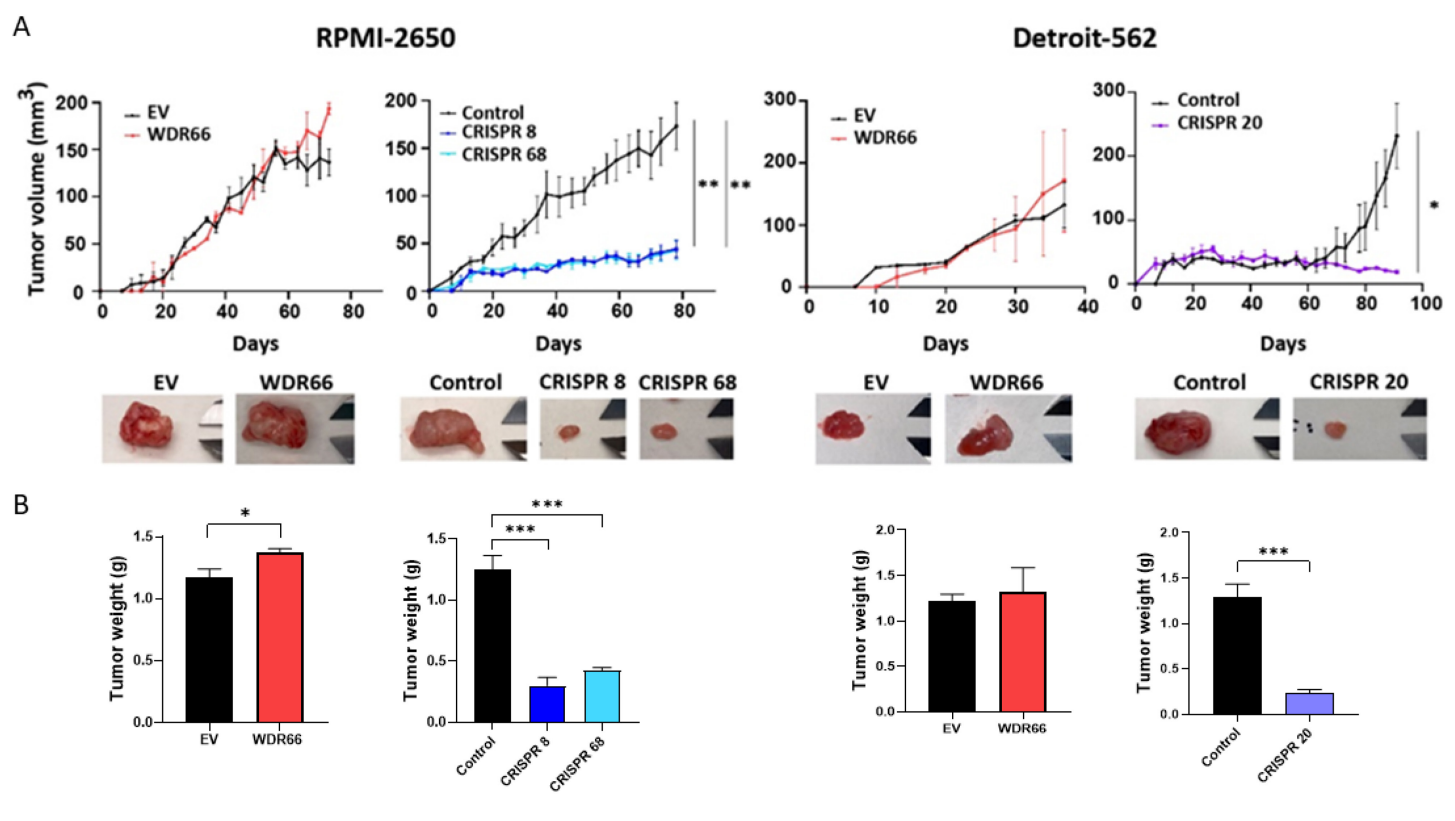
** Study of WDR66 up/downregulation in tumor growth *in vivo***. **(A)** (**Upper graphs**) Evaluation of tumor growth of xenografts generated from WDR66 up/downregulated cells in comparison to parental cell lines. Graphs represent the tumor size (mean±SEM). N=6. (**Bottom pictures**) Representative images of the tumors are shown. **(B)** Tumor weight at the experimental endpoint (day of sacrifice). Statistical analysis was performed with Student's t test (*p<0.05; **p<0.01; ***p<0.001). The absence of an asterisk means that the data are not statistically significant.

Next, we decided to validate whether WDR66 overexpression had any functional effects *in vivo*. To do so, we performed xenograft of the RPMI-2650 and Detroit-562 lines in immunosuppressed mice. The results showed no significant differences in tumor growth rates between the two conditions (**Fig. 6A**). Regarding the final size of these tumors, we observed a larger size of the tumors formed from cells with WDR66 overexpression in both cell lines and this was further supported by the measurement of tumor weight at the experimental endpoint, although there was considerable variability in size in Detroit-562 (**Fig. 6A**).

Because we had observed a reduction in the expression of genes related to the CSC phenotype and in some tumor properties when we decreased WDR66 expression, cells from the RPMI-2650 and Detroit-562 cell lines were injected into immunosuppressed mice. In both cell lines, tumors derived from cells from the CRISPR clones grew much more slowly than those from the parental cells (**Fig. 6A**). Notably, in the Detroit 562 model, tumor latency differed between the overexpression and CRISPR settings. This discrepancy can be attributed to methodological differences, as overexpression experiments were performed in bulk cultures, whereas CRISPR knockout tumors were generated from single cell-derived clones. Clonal populations are known to display intrinsic variability in tumor initiating capacity and latency, which is particularly pronounced in Detroit 562 cells. This biological variability explains the differences observed in control groups without compromising the interpretation of WDR66 dependent effects. Consistently, both tumor size and tumor weight at the endpoint were reduced in tumors derived from cells with decreased WDR66 expression compared to those generated from the parental cells. In one of the mice injected with CRISPR 8 cells, we did not obtain a tumor (**Fig. 6A-B**).

### Study of the role of WDR66 in the tumor microenvironment

**Figure 7:**
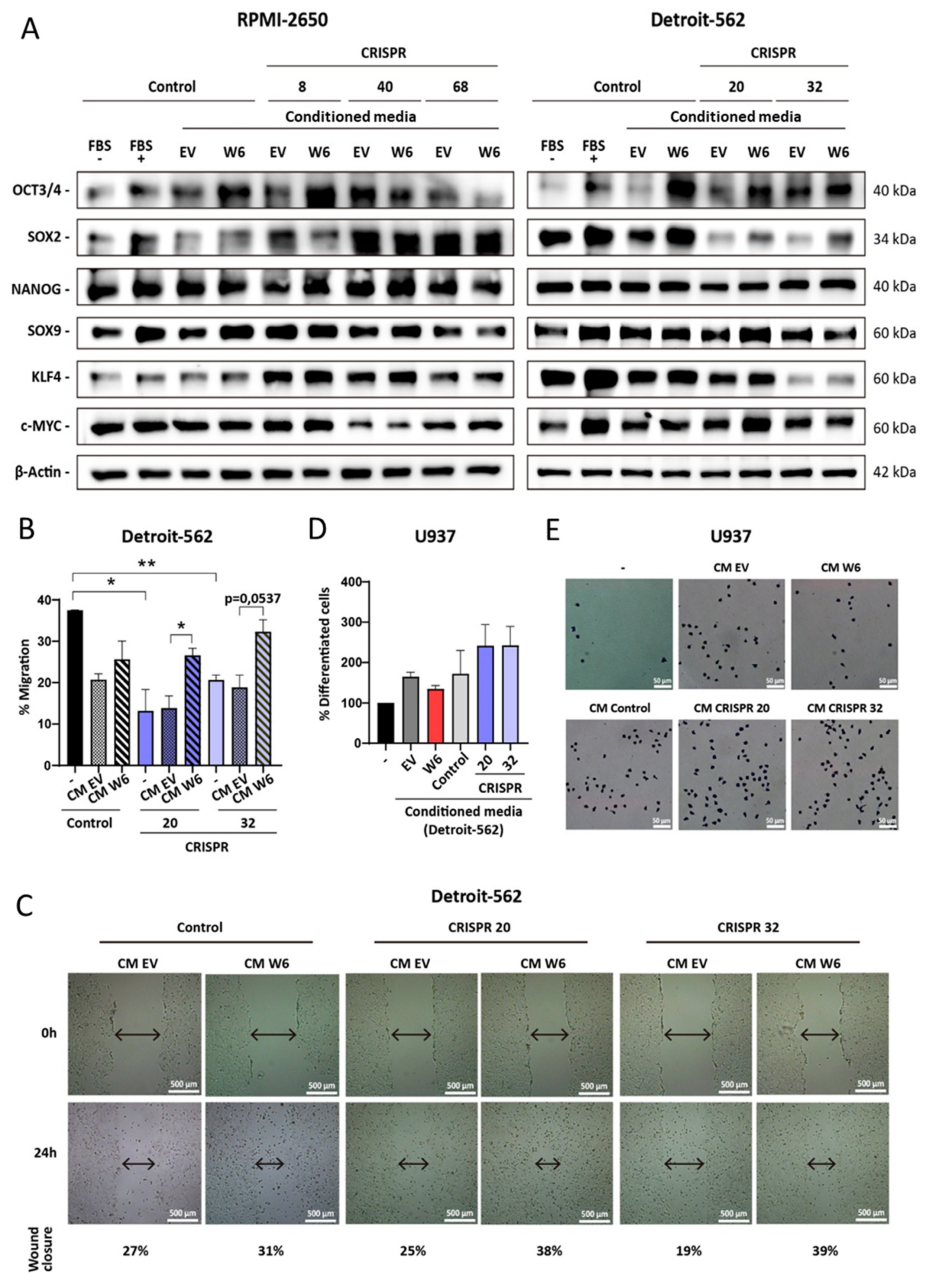
** Effect of the conditioned medium of cells with overexpression of WDR66**. **(A)** Study of the levels of proteins related to the stem cell phenotype by Western-blot after treating the parental cells and CRISPR clones with conditioned medium produced by EV cells and with overexpression of WDR66, in RPMI-2650 and Detroit-562. **(B-E)** Effect of conditioned medium of cells with overexpressed WDR66 on cell migration and differentiation. **(B)** Wound healing assay by treating parental cells and Detroit-562 CRISPR clones with conditioned medium produced by EV cells and overexpressing WDR66 (50% conditioned medium + 50% normal medium without serum). **(C)** Representative images of wound closure are shown at the beginning of the experiment and after 24 hours, and the percentages of closure over the control (0h) obtained in each case. **(D)** Differentiation assay by treating U937 cells with conditioned medium (50% MC + 50% normal medium) produced by Detroit-562 EV cells, with overexpression of WDR66, CRISPR 20 and 32. Data are represented by normalizing the condition to 100% control treated with normal medium. **(E)** Representative images are shown of the number of differentiated cells that remained adhered to the plate, after fixation and staining with crystal violet. The mean of 3 independent experiments ± SEM is presented. Statistical analysis was performed with Student's t test (*p < 0.05; **p < 0.01; ***p < 0.001). The absence of an asterisk means that the data are not statistically significant.

Another property that may contribute to clonal selection during tumor evolution is the ability of the microenvironment to support tumor growth. As is known, tumor cells communicate with different cell types, such as fibroblasts, immune system cells and endothelial cells, through the secretion of multiple factors that affect processes such as migration, immune evasion and angiogenesis. To explore this possible role of WDR66, conditioned medium (serum-free) was generated from cells with overexpression of this protein and used to treat parental cells and CRISPR clones with reduced WDR66. When Western blot analysis was performed to analyze the expression of markers associated with the stem cell phenotype, such as OCT3/4, SOX2, KLF4 and NANOG, a generalized increase in their expression was observed in cells treated with the overexpression conditioned medium, even in clones that previously showed low basal levels of these genes (**Fig. 7A and Supplementary Fig. 6**). This effect was observed in both cell lines, although with nuances between them.

This finding led to the question of whether the conditioned medium would also have an effect on cell migration. To this end, a wound healing assay was performed with Detroit-562 and its CRISPR clones, as this line had previously shown decreased migration upon losing WDR66. When treated with conditioned medium from cells with WDR66 overexpression, an increased ability to close the wound was observed, suggesting that soluble factors secreted by these cells promote migration, even in contexts of low WDR66 expression (**Fig. 7B-C**).

With these results, the impact on cell differentiation was also explored using the U937 monocytic cell line, a classic model for studying macrophage maturation. This line was treated with conditioned medium from EV cells overexpressing WDR66, and also with media from CRISPR clones with reduced WDR66. Surprisingly, the medium from the overexpressed cells reduced the number of adherent cells (indicative of decreased differentiation) (p = 0.0538), while the medium from the CRISPR clones promoted differentiation (p = 0.2252; p = 0.2003, respectively). Although the differences were not always significant, this pattern points to a possible role of WDR66 in the secretion of factors that hinder cell maturation (**Fig. 7D-E**).

**Figure 8:**
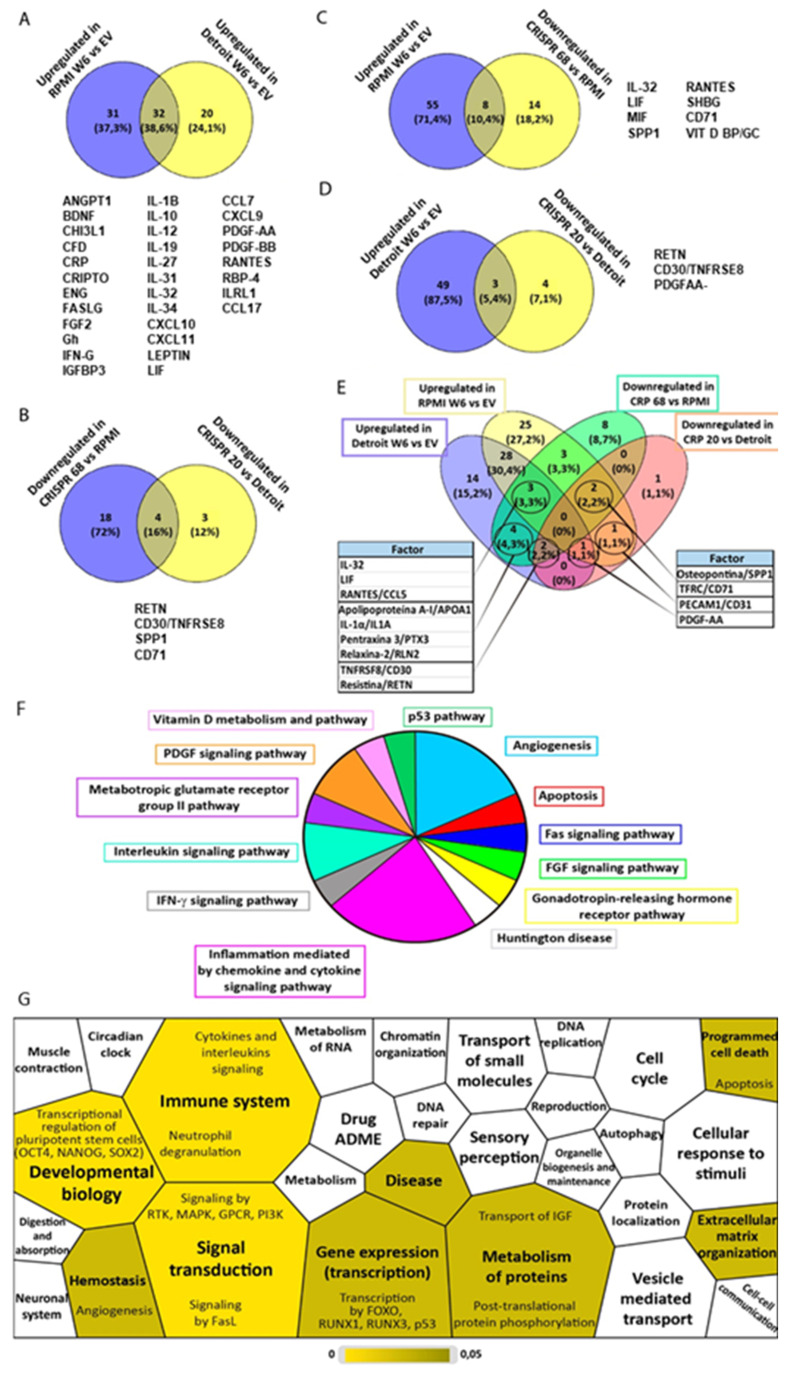
** Study of the factors present in the conditioned medium of cells with up/dowregulation of WDR66**. **(A)** Common factors increased in the conditioned medium of RPMI-2650 and Detroit-562 cells overexpressing WDR66 versus EV. **(B)** Decreased common factors in conditioned medium from CRISPR 68 cells versus RPMI-2650 and from CRISPR 20 cells versus Detroit-562. **(C)** Common factors increased in the conditioned medium of RPMI-2650 cells that overexpress WDR66 and decreased in CRISPR 68. **(D)** Common factors increased in the conditioned medium of Detroit-562 cells that overexpress WDR66 and decreased in CRISPR 20. **(E)** Common factors among the conditioned means of all conditions. **(F, G)** Study of the processes and signaling pathways in which the factors present in the conditioned medium of cells with increased and reduced expression of WDR66 participate. **(F)** Study of the processes and signaling pathways in which common soluble factors participate using the Panther tool. **(G)** Similar study performed using the Reactome tool. Those processes that were not significant appear in white, while those that did appear in yellow. The different shades of yellow indicate statistical significance, with processes in lighter yellow being the most significant.

To better understand which molecules were behind these effects, the contents of the conditioned medium were analyzed using a soluble protein array. More than 100 factors were detected, including cytokines, chemokines and growth factors. Thirty-two factors were identified as increased in the conditioned media of cells with WDR66 overexpression, and four were consistently decreased in the media of CRISPR clones (**Fig. 8A-E**). Furthermore, several factors were found to be both increased in WDR66 overexpression and decreased in WDR66 loss (**Fig. 8A-E**), reinforcing their potential functional relevance.

Bioinformatic analysis of these factors, using Panther and Reactome, revealed their involvement in signaling pathways primarily related to inflammation, angiogenesis, immune response and, to a lesser extent, apoptosis, differentiation, and developmental biology (**Fig. 8F-G**). These data suggest that WDR66 not only plays an autonomous role in tumor cells but can also modify its immediate environment through the secretion of signals that favor a more aggressive, less differentiated, and more migratory microenvironment, which could have relevant implications for tumor progression and therapy resistance.

## Discussion

In this study, we provide evidence that WDR66 is significantly overexpressed in head and neck squamous cell carcinoma (HNSCC) compared to normal tissues and that its high expression correlates with worse overall patient survival. While WDR66 expression was also observed in testicular and cervical cancers, its consistent overexpression across multiple HNSCC datasets, including primary tumors, metastases, and all clinical stages, underscores its potential as a biomarker of aggressive disease in this cancer type. These findings raise the possibility that WDR66, previously poorly characterized in oncology, may contribute to the molecular landscape of HNSCC by regulating processes associated with cancer stemness, epithelial-mesenchymal transition (EMT), apoptosis resistance, immune microenvironment and therapy resistance.

Interestingly, functional analyses revealed that WDR66 does not directly modulate proliferation or clonogenicity, which distinguishes it from canonical oncogenes such as MYC or CCND1 that drive HNSCC proliferation 35. Instead, WDR66 appears to influence subtler but equally critical aspects of tumor biology, such as cellular plasticity, survival under stress, and tumor adaptability. This suggests that WDR66 may act as a “non-classical oncogenic facilitator”, a regulator of cellular states that enables tumor persistence and progression under therapeutic or microenvironmental pressures.

Our data demonstrate that WDR66 enhances stemness-associated traits, including tumorsphere formation, upregulation of pluripotency transcription factors (notably OCT4 and SOX2), and suggest a cell line-dependent modulation of CSC surface markers (CD10, CD184). This phenotype is reminiscent of transcriptional reprogramming processes and overlaps with pathways activated during induced pluripotency. It should be noted that overexpression experiments were performed on bulk transfected populations, whereas CRISPR mediated knockdown required the use of single cell-derived clones. Bulk cultures reduce biological heterogeneity, while single clones may retain intrinsic variability unrelated to WDR66 editing. This methodological difference may partly explain the subtle discrepancies observed between the two models, particularly regarding CD184 and CD10 expression in Detroit 562 cells. Indeed, WDR66 increased the number of iPSC colonies obtained in a fibroblast reprogramming assay, although the effect did not reach statistical significance. These results suggest that WDR66 may act as a permissive factor for pluripotency by modulating chromatin structure or interacting with stemness regulators. Given that WDR66 is a WD-repeat protein, one plausible hypothesis is that it functions as a scaffolding protein facilitating multiprotein complex assembly, similar to other WD-repeat proteins 36, 37. Such scaffolding roles could position WDR66 as a regulator of transcription factor activity, epigenetic modifiers, or RNA-binding proteins relevant to stemness.

A particularly striking finding was the consistent upregulation of LIN28A/B in WDR66-overexpressing cells, suggesting that WDR66 may regulate stemness through LIN28/let-7 signaling. Since LIN28 inhibits let-7 microRNAs, which are well-established tumor suppressors 38, 39, WDR66 overexpression could tip the balance toward self-renewal and dedifferentiation, while WDR66 downregulation may restore let-7 activity, leading to differentiation and loss of stemness. This mechanistic link would explain why WDR66-high tumors exhibit poorer survival: they may harbor a persistent CSC compartment resistant to therapy. Similar connections between stemness regulators and therapy resistance have been observed in HNSCC, where ALDH1, SOX2, and NANOG sustain CSC populations and promote recurrence 40, 41. Our data suggest that WDR66 should now be considered within this network.

In addition to stemness, WDR66 altered the expression of EMT-associated genes, including TWIST1, SNAI1, VIM, and FOXC2, in a cell line-dependent manner. While the migratory phenotype was most pronounced in Detroit-562 cells, the transcriptional changes observed across models suggest that WDR66 promotes partial EMT programs, a state increasingly recognized as central to metastasis and CSC maintenance 42. Of note, the PCR array identified SPP1 and SNAI3 as consistently upregulated, both of which are implicated in invasive behavior and metastatic potential 43, 44. These findings raise the possibility that WDR66 drives a hybrid stemness/EMT state, enabling both self-renewal and migratory capacity, which could underlie its association with metastasis in patient data.

Another major observation was that WDR66 protects HNSCC cells from apoptosis and necrolysis. Overexpression reduced basal apoptosis, suppressed PARP1 cleavage, and downregulated caspases, while CRISPR-mediated knockdown increased apoptotic activation. This anti-apoptotic effect extended to chemotherapy resistance, as WDR66-overexpressing cells were more resistant to cisplatin and paclitaxel, while knockdown sensitized cells to cisplatin and (to a lesser extent) 5-fluorouracil. The connection between CSC-like phenotypes and drug resistance is well-established 45, and our data suggest that WDR66 contributes to this axis by inhibiting apoptosis while sustaining stemness programs. Mechanistically, this could occur through transcriptional regulation of survival pathways (e.g., PI3K/AKT, NF-κB, or STAT3), which are also implicated in CSC maintenance in HNSCC 6. Given that WDR66 overexpression reduced apoptotic sensitivity most strongly in Detroit-562 cells, cell context likely dictates which signaling nodes WDR66 interfaces with.

The *in vivo* xenograft data further support WDR66's role as a facilitator of tumor aggressiveness. While overexpression did not significantly alter growth kinetics, tumors from WDR66-overexpressing cells were larger, whereas knockdown reduced tumor size and, in some cases, completely abrogated tumor formation. These findings suggest that WDR66 is not required for basal proliferation but is critical for tumor initiation and maintenance, consistent with its proposed function in sustaining CSC populations. The fact that CRISPR-mediated depletion yielded incomplete penetrance of tumor formation highlights its potential as a therapeutic vulnerability in HNSCC.

Taken together, our findings position WDR66 as a key regulator of plasticity and survival in HNSCC. Rather than driving proliferation, WDR66 appears to orchestrate a network of pathways that promote CSC phenotypes, EMT, apoptosis resistance, and therapy resistance. These attributes converge to produce tumors that are more aggressive, metastatic, and refractory to treatment.

Future work should aim to elucidate the molecular interactome of WDR66, particularly its relationship with LIN28, let-7 microRNAs, and EMT transcription factors. Proteomic or chromatin immunoprecipitation studies could identify whether WDR66 acts as a scaffolding protein in stemness-related transcriptional complexes or whether it regulates post-transcriptional RNA metabolism. Moreover, investigating whether WDR66 expression is regulated by oncogenic signaling pathways such as EGFR/PI3K/AKT or Wnt/β-catenin may provide insight into its integration into broader oncogenic networks. Finally, given its correlation with poor prognosis, WDR66 expression could be developed as a prognostic biomarker to stratify HNSCC patients for aggressive therapy or clinical trial enrollment. Therapeutically, targeting WDR66 directly, or its downstream effectors such as LIN28, may offer novel strategies to overcome CSC-driven resistance and improve patient survival.

In summary, our results indicate that WDR66 influences several features associated with stemness and pluripotency in HNSCC cells, although in a cell line dependent manner, likely reflecting differences in basal WDR66 expression levels (Supplementary Fig. 7). WDR66 overexpression promotes tumorsphere formation and upregulates key pluripotency related transcription factors and regulators such as LIN28, whereas WDR66 downregulation consistently reduces tumorsphere size and decreases CSC associated surface markers, particularly in Detroit 562. Nonetheless, some effects appear as trends rather than statistically significant changes, and not all stemness markers behave uniformly across the two models. Together, these findings support a potential role for WDR66 in modulating stemness related programs in HNSCC, while highlighting the complexity and context-dependence of this regulation.

### WDR66 in the microenvironment

The tumor microenvironment is a complex environment where cancer cells interact with neighboring cells such as fibroblasts, endothelial cells, and immune cells. All of these interactions modulate key aspects of the tumor, such as its growth, migration, invasion, and resistance to treatment therapies 46-48. In this context, cells that overexpress WDR66 secrete factors that promote a stem cell phenotype and increase cell migration, even in cells with low levels of WDR66, suggesting that these secreted proteins influence tumor aggressiveness. Furthermore, conditioned medium from cells with high WDR66 expression tends to inhibit monocyte-to-macrophage differentiation, while that produced by cells with low WDR66 expression appears to promote it, pointing to a possible role for WDR66 in immunomodulation through secreted factors.

By studying the soluble factors secreted by cells with different levels of WDR66, multiple proteins involved in tumor growth and progression were detected, such as CRIPTO, IGFBP3, and PDGF, which are increased when WDR66 is overexpressed. These factors can activate signaling pathways that promote proliferation, migration, angiogenesis, and resistance to apoptosis 49-53. CRIPTO, in particular, stood out for being increased at both the RNA and secreted protein levels, reinforcing its potential importance in the effect that WDR66 has on tumor cells. Furthermore, IGFBP3 could help cells survive adverse conditions, such as exposure to chemotherapy, while PDGF, known to attract stromal cells and increase resistance to treatment, is also elevated and could contribute to a worse prognosis 50-52. Furthermore, the influence of WDR66 and the factors it modulates varies by cell line, reflecting differences depending on the tumor origin (nose or pharynx). Taken together, these data suggest that WDR66 not only directly affects tumor cells but also modifies their microenvironment by secreting factors that enhance a more aggressive, less differentiated, and more therapy-resistant phenotype, which could partly explain cancer progression and aggressiveness in a more realistic physiological.

In our study, we observed a significant increase in several cytokines, low-molecular-weight proteins that regulate processes such as proliferation, survival, migration, and immune system activation 54, 55. These cytokines, secreted by tumor cells or the immune system, can generate feedback loops that promote both antitumor and protumor effects, coexisting in different phases of the disease. Among the dysregulated cytokines are IL-31, which is associated with tumor stage and metastasis, and IFN-γ, which has both antitumor and tumor progression-promoting functions. Chemokines such as CCL7, linked to cell migration and invasion, and CCL5, which activates pathways that facilitate tumor progression and is associated with a worse prognosis, were also increased. Many cytokines activate or induce the production of others, such as IL-31, which stimulates the secretion of CCL1, CCL22, and CCL17, or IFN-γ, which increases the expression of receptors and cytokines such as CXCL10 and CXCL11. Osteopontin/SPP1 stands out, promoting proliferation, angiogenesis, and metastasis, and is related to other cytokines increased in the study 43, 56, 57. We also identified the cytokine LIF, involved in the regulation of cellular pluripotency and tumor progression, which could be linked to the increase in pluripotent stem cells induced by overexpressing WDR66 50. Soluble receptors such as IL1RL1/ST2, which modulates the immune response and is associated with tumor progression through macrophage polarization, were also found 58.

On the other hand, an increase in FASL/CD95L, involved in immune evasion and pro-tumor signaling 59, and TNFRSF8/CD30, a receptor associated with tumor progression and immune resistance, were observed 60, 61. In addition, we detected multiple soluble factors related to the immune system, angiogenesis, metabolism, and molecular transport, which together regulate processes such as immune signaling, programmed cell death, and the organization of the extracellular matrix. Some normally membrane-bound proteins could be secreted in soluble forms or in extracellular vesicles under our experimental conditions. WDR66, for its part, is involved in the ciliary structure and the cytoskeleton, which could explain the changes in cell migration observed when modifying its expression, possibly also affecting vesicular trafficking and the secretion of factors into the tumor microenvironment 62-64. In summary, cells with WDR66 overexpression secrete factors that promote migration and dedifferentiation, while WDR66 reduction decreases migration and growth.

Currently, attempts are being made to correlate the cytokine profile in biological fluids with the response to therapies, especially immunotherapies, considering cytokines as potential molecular biomarkers. However, studying individual cytokines does not usually capture the complexity of the tumor microenvironment, so identifying a minimal signature could help personalize treatments and predict responses. In recurrent or metastatic head and neck cancer (HNSCC), immune checkpoint inhibitors (anti-PD1) are the first line of treatment, being most effective in "inflamed" tumors with cytotoxic T lymphocyte infiltration and effector cytokine secretion 46, 65, 66. Despite this, the clinical benefit is modest, and there is still no clear predictive cytokine pattern due to the heterogeneity of HNSCC. Furthermore, the microenvironment varies depending on whether the tumor is HPV-positive or -negative. HPV+ tumors have greater immune infiltration and a better response to immunotherapy, while HPV- tumors show less infiltration and a worse prognosis, although those with high immune infiltration also respond better 67, 68. In our study, the cell lines were HPV-, but WDR66 overexpression increased the secretion of many cytokines, suggesting that it may contribute to a more inflamed tumor microenvironment, potentially associated with greater immune infiltration. Since WDR66 is associated with a poor prognosis and tumor progression, we propose that it could potentially serve as a biomarker of response to immunotherapy in head and neck cancer, although this would require further validation in appropriate *in vivo* models.

In summary, WDR66 influences tumor biology intracellularly, affecting pluripotency, migration, resistance to therapies, apoptosis and tumor growth, in addition to promoting the secretion of soluble factors that modulate the microenvironment, favoring tumor progression. This makes it a potential biomarker of both poor prognosis and response to immunotherapy in HNSCC.

## Materials and Methods

### Cell culture

RPMI-2650 (RRID:CVCL_1664) and Detroit-562 (RRID:CVCL_1171) cell lines were obtained from the commercial repository ECACC. No further authentication was done by the authors. Cells tested negative for mycoplasma. RPMI-2650 and Detroit-562 cell lines and MEFs (mouse embryonic fibroblasts) were maintained as indicated in 47. Feeder leyer (SNL) cells (RRID:CVCL_K227) were grown and maintained in DMEM (Gibco) supplemented with 15% fetal bovine serum (FBS) (Gibco), penicillin and streptomycin (Sigma). Cells were periodically tested for mycoplasma, all negative.

### Retroviral infection

Retroviral vectors and gene transfer were performed as previously described in 69.

### CRISPR/Cas9 to generate WDR66 knockout

An sgRNA targeting the WDR66 sequence CACAGCGTCCATGATCCGTT (exon 2) was used to generate the knockdown. First, we infected the cells with virus containing WDR66-sgRNA and drug selection. Then, the cells were isolated by single-cell sorting by FACS Jazz (BD Biosciences) in 96-well plates 70. One month later, each well that grew was amplified and validated by Western blot analysis. The selected CRISPRs were sequenced by the Genomics and Sequencing service at IBiS.

### RT‒qPCR

Total RNA was extracted and purified using the ReliaPrepTM RNA Tissue Miniprep System from Promega. We used 3 µg of mRNA for reverse transcription by using the High-Capacity cDNA Reverse Transcription kit (Life Technologies) according to the manufacturer's instructions. We used the following specific probes as indicated in **Table 1**.

### Protein isolation and western blot analysis

Western blots were performed as previously described elsewhere 71. Membranes were incubated with primary antibodies as indicated in **Table 2**.

The proteins were detected using an ECL detection system (Amersham Biosciences) and a Bio-Rad Chemidoc Touch.

### Growth curve

To measure the proliferation capacity, 2.5x10^3^ (RPMI-2650) or 8x10^3^ (Detroit-562) cells were seeded in 12-well plates in triplicate and procedure followed as in 47. Briefly, at 24 h (Day 0), and then a point for the growth curve each 48hrs cells was fixed with 0.5% glutaraldehyde up to 10 days. Then, the plates were stained with 1% crystal violet (Sigma). Later, the crystal violet was dissolved with 20% acetic acid (AppliChem), and the relative number of cells was quantified by measuring the absorbance of the crystal violet at 595 nm (Bio-Rad). The values are presented relative to Day 0.

### Clonogenic assay

To measure individual clones fromed by the cell lines, 500 (RPMI-2650) or 5x10^3^ (Detroit-562) cells were plated in 10 cm plates in triplicate. Cells were fixed with 0.5% glutaraldehyde and stained with 1% crystal violet after 15 days. The number of colonies was counted, and types of clones were classified.

### Tumorsphere assay

A total of 1×10^4^ (RPMI-2650) or 2×10^4^ (Detroit-562) cells were seeded in triplicate in 24-well Ultra-Low Attachment Plates (Costar). Media contains 1 mL of MammoCult basal medium (Stem Cell Technologies) supplied with 10% MammoCult proliferation supplement: 4 μg/mL heparin, 0.48 μg/mL hydrocortisone, penicillin and streptomycin. After 5-10 days, depending on the cell line, the number of primary tumorspheres formed was measured.

### Fluorescence-activated cell sorting (FACS) analysis

For FACS analysis, 1x10^6^ cells were trypsinized and suspended in 100 µL of PBS containing 2% FBS and 5 mM EDTA. Cells were blocked with 10 µL of human blocking reagent (Miltenyi Biotec) for 10 min at 4 °C. Then, the cells were incubated with 2 µL of anti-CD10-FITC (Miltenyi Biotec #130-124-215) or 2 µL of anti-CD184-PE (Miltenyi Biotec #130-117-690) for 30 min at 4 °C. After washing the cells twice with PBS-FBS-EDTA, they were suspended in 500 µL of the same buffer and analyzed by FACS with the FACS Canto II cytometer (BD Biosciences). Experiments were repeated a minimum of three times independently in triplicate samples.

### Cell death assay

Apoptosis was evaluated using the Annexin V-FITC/PI kit (Inmunostep). Cells were stained and categorized as early/late apoptotic or necrotic via flow cytometry (FACS Canto II). Controls included unstained, Annexin-only, and PI-only samples.

### Wound healing migration assay

To evaluate cell migration, a wound healing assay was performed. Cells were seeded at 80-90% confluence in 6-well plates (duplicates), with two parallel guide lines marked underneath. The next day, a scratch ("wound") was made perpendicular to the lines using a pipette tip, washed with PBS, and medium without FBS was added. For conditioned medium experiments, 50% of the medium was replaced with conditioned media from Detroit-562 EV/WDR66 cells. This media was collected after 24 h of serum-free culture, filtered (0.45 µm), and applied to scratched cells. Images were taken at 0 h and every 3-4 h up to 28 h using an Olympus IX-71 microscope. Wound closure was quantified using Adobe Photoshop 2020.

### Cytotoxicity assay

Cytotoxicity was assessed in 96-well plates using 6,000 RPMI-2650, 20,000 Detroit-562 cells per well. After 24 h, cells were treated with a range of concentrations of cisplatin (50-0.25 µM), paclitaxel (2-1.69x10⁻⁵ µM), and 5-fluorouracil (200-1 µM). After 96 h, cells were fixed and stained with 0.5% crystal violet. Absorbance was measured at 595 nm after solubilizing the dye with 20% acetic acid. IC₅₀ values were calculated by comparing to untreated controls.

### Monocyte to macrophage differentiation assay

100,000 U937 cells were seeded in 6-well plates. Conditioned medium (50%) from Detroit-562 variants (EV/WDR66, CRISPR clones) was added after filtering. Control cells received standard medium. After 72 h, non-adherent cells were washed off, and adherent cells were fixed with 0.5% glutaraldehyde, stained with 0.5% crystal violet, and visualized under a microscope. Staining intensity was quantified by solubilizing the dye in 20% acetic acid and measuring absorbance at 595 nm.

### PCR array

To evaluate gene expression changes related to stem cell phenotype and epithelial-to-mesenchymal transition (EMT), PCR arrays were performed in cells overexpressing WDR66.

### Stem cell phenotype gene analysis

A pre-designed PCR array card from Applied Biosystems (Thermo Fisher Scientific, Ref: 4385344) was used, containing the following: Controls: ACTB, RAF1, CTNNB1, GAPDH, EEF1A1, and 18S. Pluripotency-related genes: NANOG, POU5F1, SOX2, TDGF1, DNMT3B, GABRB3, GDF3. Other stemness-associated genes: BRIX, CD9, COMMD3, CRABP2, EBAF, FGF4, FGF5, FOXD3, GAL, GBX2, GRB7, IFITM1, IFITM2, IL6ST, IMP2, KIT, LEFTB, LIFR, LIN28, NODAL, NOG, NR5A2, NR6A1, PODXL, PTEN, REST, SEMA3A, SFRP2, TERT, TFCP2L1, UTF1, XIST, ZFP42. Differentiation markers: ACTC, AFP, CD34, CDH5, CDX2, CGB, COL1A1, COL2A1, DDX4, DES, EOMES, FLT1, FN1, FOXA2, GATA4, GATA6, GCG, GCM1, GFAP, HBB, HBZ, HLXB9, IAPP, INS, IPF1, ISL1, KRT1, LAMA1, LAMB1, LAMC1, MYF5, MYOD1, NES, NEUROD1, NPPA, OLIG-2, PAX4, PAX6, PECAM1, PTF1A, RUNX2, SERPINA1, SOX17, SST, SYCP3, SYP, T, TAT, TH, WT1.

### EMT gene analysis

For EMT-related gene expression, another PCR array card from Applied Biosystems (Thermo Fisher Scientific, Ref: 4391016) was used. It included: Controls: 18S, GAPDH, GUSB, HPRT1, ILR1N EMT-related genes: ACTB, AHNAK, AKT1, B2M, BMP1, BMP7, CALD1, CAMK2N1, CAV2, CDH1, CDH2, COL3A1, COL5A2, CTNNB1, DESI1, DSC2, DSP, EGFR, ERBB3, F11R, FGFBP1, FN1, FOXC2, FZD7, GEMIN2, GNG11, GSC, GSK3B, HMBS, IGFBP4, ILK, ILR1N, IPO8, ITAG5, ITGAV, ITGB1, JAG1, KRT19, KRT7, MAP1B, MITF, MMP2, MMP3, MMP9, MSN, MST1R, NOTCH1, NUDT13, OCLN, PDGFRB, PGK1, PLEK2, PTK2, PTP4A1, RAC1, RGS2, RPLP0, SERPINE1, SMAD2, SNAI1, SNAI2, SNAI3, SPARC, SPP1, STAT3, STEAP1, TBP, TCF3, TCF4, TFPI2, TFRC, TGFB1, TGFB2, TGFB3, TIMP1, TMEFF1, TMEM132A, TSPAN13, VCAN, VIM, VPS13A, WNT11, WNT5A, WNT5B, ZEB1, ZEB2.

### Data analysis

The results were visualized using Venn diagrams created with the online tool from the National Center for Biotechnology (CNB-CSIC): https://bioinfogp.cnb.csic.es/tools/venny/.

### Soluble factor array

The relative levels of various soluble factors were analyzed in cells with overexpression or downregulation of WDR66 using the Proteome Profiler Human XL Cytokine Array Kit (ARY022B, Bio-Techne). This kit includes antibodies against a wide panel of cytokines, chemokines, growth factors, and other soluble molecules: Adiponectin, Apolipoproteín A-I, Angiogenin, Angiopoietin-1, Angiopoietin-2, BAFF, BDNF, C5/C5a, CD14, CD30, CD40 Ligand, Quitinase 3-*like*, Complement factor D, Proteín C-Reactive, Cripto-1, Cistatin C, Dkk-1, DPPIV, EGF, EMMPRIN, ENA-78, Endoglin, Fas Ligand, FGF basic, FGF-7, FGF-19, Flt-3 Ligand, G-CSF, GDF-15, GM-CSF, GROα, Growth hormone, HGF, ICAM-1, IFN-γ, IGFBP-2, IGFBP-3, IL-1α, IL-1β, IL-1ra, IL-2, IL-3, IL-4, IL-5, IL-6, IL-8, IL-10, IL-11, IL-12 p70, IL-13, IL-15, IL-16, IL-17A, IL-18 Bpa, IL-19, IL-22, IL-23, IL-24, IL-27, IL-31, IL-32, IL-33, IL-34, IP-10, I-TAC, Kallikreín 3, Leptin, LIF, Lipocaline-2, MCP-1, MCP-3, M-CSF, MIF, MIG, MIP-1α/MIP-1β, MIP-3α, MIP-3β, MMP-9, Mieloperoxidase, Osteopontin, PDGF-AA, PDGF-AB/BB, Pentraxin 3, PF4, RAGE, RANTES, RBP-4, Relaxina-2, Resistin, SDF-1α, Serpin E1, SHBG, ST2, TARC, TFF3, TfR, TGF-α, Thrombospondin-1, TNF-α, uPAR, VEGF, Vitamin D BP, CD31, TIM-3, VCAM-1.

Cells were cultured in serum-free medium for 48 hours with 4 mL of medium per dish. The collected medium was filtered through a 0.2 µm filter to remove cell debris, and the assay was performed according to the manufacturer's instructions: Blocking: 2 mL of blocking buffer (buffer 6) was added to the assay plate wells containing the membranes, incubated for 1 hour with agitation. Sample incubation: 500 µL of conditioned medium was diluted in 1 mL of blocking buffer and added to the membranes. Membranes were incubated overnight at 4 °C with agitation. Washing: Membranes were washed three times with 1x wash buffer. Detection antibody mix: 30 µL of detection antibody cocktail was diluted in 1.5 mL of buffer 4/6 (4 mL buffer 4 + 8 mL buffer 6) and added to each membrane, incubated for 1 hour at room temperature with agitation. Streptavidin-HRP incubation: After further washes, streptavidin-HRP (1:2000 dilution in buffer 6) was added for 30 minutes. Signal detection: Membranes were washed again, developed with 1 mL of detection reagent, and visualized using Chemidoc Touch (Bio-Rad). Analysis: Signal quantification was performed using Image Lab software. Results were visualized using Venn diagrams via the CNB-CSIC online tool: https://bioinfogp.cnb.csic.es/tools/venny/.

### iPS generation assay

iPS was generated as described in 47. Transfection of PhoenixE is performed with 80 μl PEI and 10 μg of each of the plasmids pMXs (Oct3/4, Sox2, Klf4, c-Myc, pCMV-EV, pCMV-WDR66 and DsRed, this last one as a transfection control) and incubated for 20 hours. On the other hand, transfection of HEK293-T cells was performed with 2 μg of pMD2G, 8 μg of psPAX2 and 10 µg of pGreenZeo mNanog plasmid. After 24 hours, medium from retrovirus- and lentivirus-producing cells was collected and filtered using a 0.45 µm acetate-cellulose filter. Polybrene was added to a final concentration of 4 µg/ml. Required combination of plasmids was added to MEFs previously seeded in a 6-well plate at 10^5^ cells per well. The cells were incubated for 24 hours, and the medium was changed. After 72 hours, 1700 infected MEFs were plated in 6-well plates containing a feeder layer of mitomycin-C-inactivated SNL cells (350,000 cells per plate). The next day, embryonic stem cell culture medium (ES medium) was added to the wells. Eight days after the first iPS cell appeared, we performed the alkaline phosphatase assay. On Day 21, we counted Nanog-GFP-positive colonies under a fluorescence microscope.

### Alkaline phosphatase assay (ALP)

We used an alkaline phosphatase detection kit (Sigma), which contains two reagents: Fast Red Violet solution (FVR) (0.8 g/L stock) and naphthol AS-BI phosphate solution (4 mg/mL) in AMPD buffer (2 mol/L), pH 9.5. First, cells were fixed with paraformaldehyde in 4% PBS for 1 minute and rinsed with PBS-0.1% Tween. The mixture of reagents in the ratio 2(FVR):1(Naphthol):1(Water) was added to the wells and incubated for 15 minutes in the dark. The cells were washed with PBS-0.1% Tween, and the pink colonies were observed under an inverted microscope.

### Xenograft in nude mice

Cells were suspended in 50 µL of Matrigel (Corning) prior to the injection. We inject subcutaneously 3×10^6^ RPMI-2650 cells or 2×10^6^ Detroit-562 into the right flanks of 4-week-old female athymic nude mice. Animals were examined weekly until the tumor size was approximately 1700 mm^3,^ and mice were sacrificed. Tumors were extracted and stored at -80 °C. Tumor volume (mm^3^) was measured using calipers. All animal experiments were performed according to the experimental protocol approved by the IBIS and HUVR Institutional Animal Care and Use Committee (0309-N-15).

### Bioinformatics and data analysis

We performed a comprehensive bioinformatic analysis integrating multiple public patient datasets from the R2 Genomics Analysis and Visualization Platform (http://hgserver1.amc.nl), canSAR and DriverDBv4. Expression levels of WDR66 were evaluated across tumor and normal tissues, as well as among different tumor stages, using data from TCGA-HNSCC and GSE30784. For the GSE30784 dataset, differential expression analysis was performed using a moderated t-test (limma) with Benjamini-Hochberg correction, while comparisons in the TCGA-HNSCC dataset (DriverDBv4) were assessed using the Wilcoxon rank-sum test. For multi-group comparisons across tumor stages (canSAR), Kruskal-Wallis testing followed by Dunn-type post hoc testing was applied. Statistical differences between tumor and normal samples were determined (p < 0.05 *, p < 0.01 **, p < 0.001 ***). Survival analysis was performed using SurvExpress, generating Kaplan-Meier curves to assess overall survival based on high and low WDR66 expression groups in the HNSC-TCGA cohort, using the log-rank (Mantel-Cox) test and Cox proportional hazards modeling.

To analyze the soluble factors identified in the Soluble Factor Array assays, we performed functional enrichment analyses using the Panther and Reactome platforms to identify biological processes and signaling pathways associated with commonly regulated soluble factors, thereby providing insights into their potential molecular roles in tumor progression.

### Statistical analysis

Statistical analyses of experiments were performed using GraphPad Prism (6.01 for Windows). Control samples, overexpression of WDR66 and the CRISPRs of WDR66 were compared using the unpaired Student's t test or Student's t test with Welch's correction, as appropriate. Experiments were performed a minimum of three times independently and in triplicate samples. P values less than 0.05 were considered statistically significant and were represented according to the following classification: p <0.05 (*), p <0.01 (**), and p <0.001 (***).

## Supplementary Material

Supplementary figures.

## Figures and Tables

**Table 1:** TaqMan probes employed in this study, including target gene, assay ID and probe reference.

	Gene	Probe
	*WDR66*	Hs.PT.58.38946576 (IDT)
EM genes	*FOXC2*	Hs00270951_s1 (Thermo)
	*SNAI1*	Hs00195591_m1 (Thermo)
	*TWIST1*	Hs01675818_s1 (Thermo)
	*VIM*	Hs00185584_m1 (Thermo)
	*CDH1*	Hs01023894_m1 (Thermo)
	*CDH2*	Hs00983056_m1 (Thermo)
	*β-catenin*	Hs00355049_m1 (Thermo)
	*FN1*	Hs.PT.58.40005963 (IDT)
PCR array	*LIN28A*	Hs.PT.58.24268123 (IDT)
	*LIN28B*	Hs.01013729 (Thermo)
	*RUNX2*	Hs.PT.56.a.19568141 (IDT)
	*FOXD3*	Hs.PT.58.26279783 (IDT)
	*TDGF1*	Hs.PT.58.39131775 (IDT)
	*TERT*	Hs.PT.58.27489922 (IDT)
Control	*GAPDH*	Hs03929097_g1 (Thermo)

IDT: Integrated DNA Technologies; Thermo: Thermo Fisher Scientific

**Table 2:** List of primary and secondary antibodies used in western blotting, immunofluorescence, and flow cytometry experiments.

Type	Protein	Brand	Dilution
Primary	WDR66	Novus Biologicals (H00144406-B01P)	1:500 (WB)
	WDR66	Novus Biologicals (NBP1-82711)	1:500 (IF) 1:200 (FC)
	DDK-tag	Origene (TA150014)	1:1000 (WB)
	OCT3/4	Santa Cruz (sc-5279)	1:200 (WB)
	SOX2	Santa Cruz (sc-365823)	1:200 (WB)
	NANOG	Santa Cruz (sc-293121)	1:200 (WB)
	SOX9	Abcam (ab185230)	1:1000 (WB)
	KLF4	Abcam (ab72543)	1:1000 (WB)
	c-MYC	Cell Signaling (5605)	1:1000 (WB)
	LIN28	Santa Cruz (sc-374460)	1:1000 (WB)
	VIM	Santa Cruz (sc-6260)	1:1000 (WB)
	CDH1	Santa Cruz (sc-8426)	1:200 (WB)
	CDH2	Santa Cruz (sc-271386)	1:200 (WB)
	PARP1	Cell Signaling (9532)	1:1000 (WB)
	Caspasa 3	Cell Signaling (9664)	1:1000 (WB)
	β-actin	Abcam (ab8226)	1:2000 (WB)
	HSP70	Abcam (ab45133)	1:1000 (WB)
Secondary	Anti-rabbit HRP	Abcam (ab97051)	1:5000 (WB)
	Anti-mouse HRP	Abcam (ab97046)	1:5000 (WB)
	Anti-rabbit Alexa Fluor 488	Invitrogen (A11008)	1:250 (IF) 1:200 (FC)

WB: Western-blot; IF: Immunofluorescence; FC: Flow cytometry

## Data Availability

All data and resources are available upon reasonable request.
