# Supplementary material for: Role of WDR66 in Stemness, Therapy Resistance and Tumor microenvironment modulation in Head and Neck Cancer
Source: Int J Biol Sci. 2026 May 22;22(11):5685–705. doi: 10.7150/ijbs.130010 (PMC13282750; doi:10.7150/ijbs.130010)
Supplement: Supplementary file 1 — Supplementary figures. [file ijbsv22p5685s1.pdf]

Supplementary Material

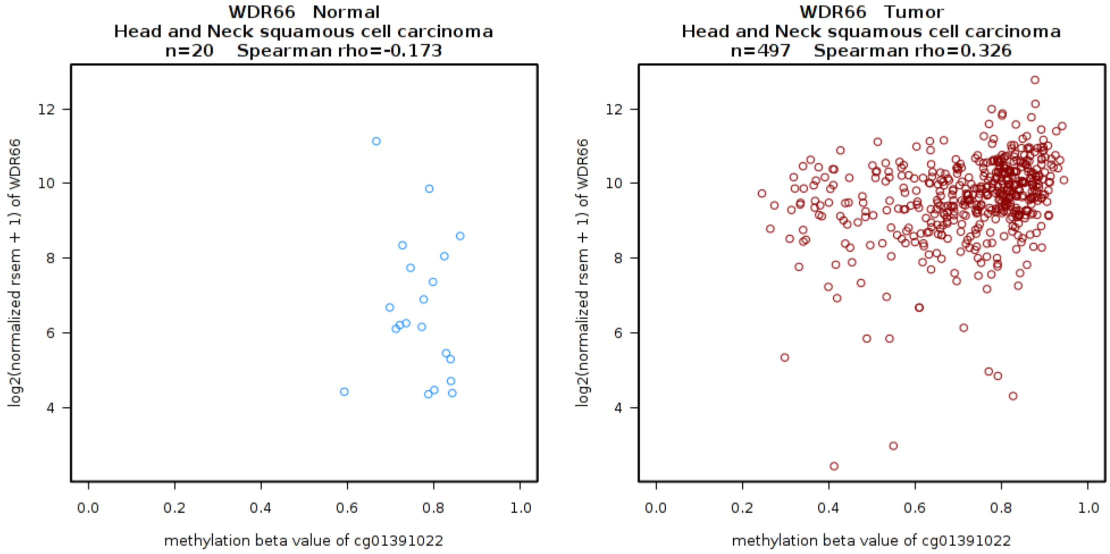

Supplementary Figure 1. *DNA methylation levels of WDR66 in normal (left) and tumor (right) tissues from HNSCC. The plot shows log-transformed methylation beta values.*

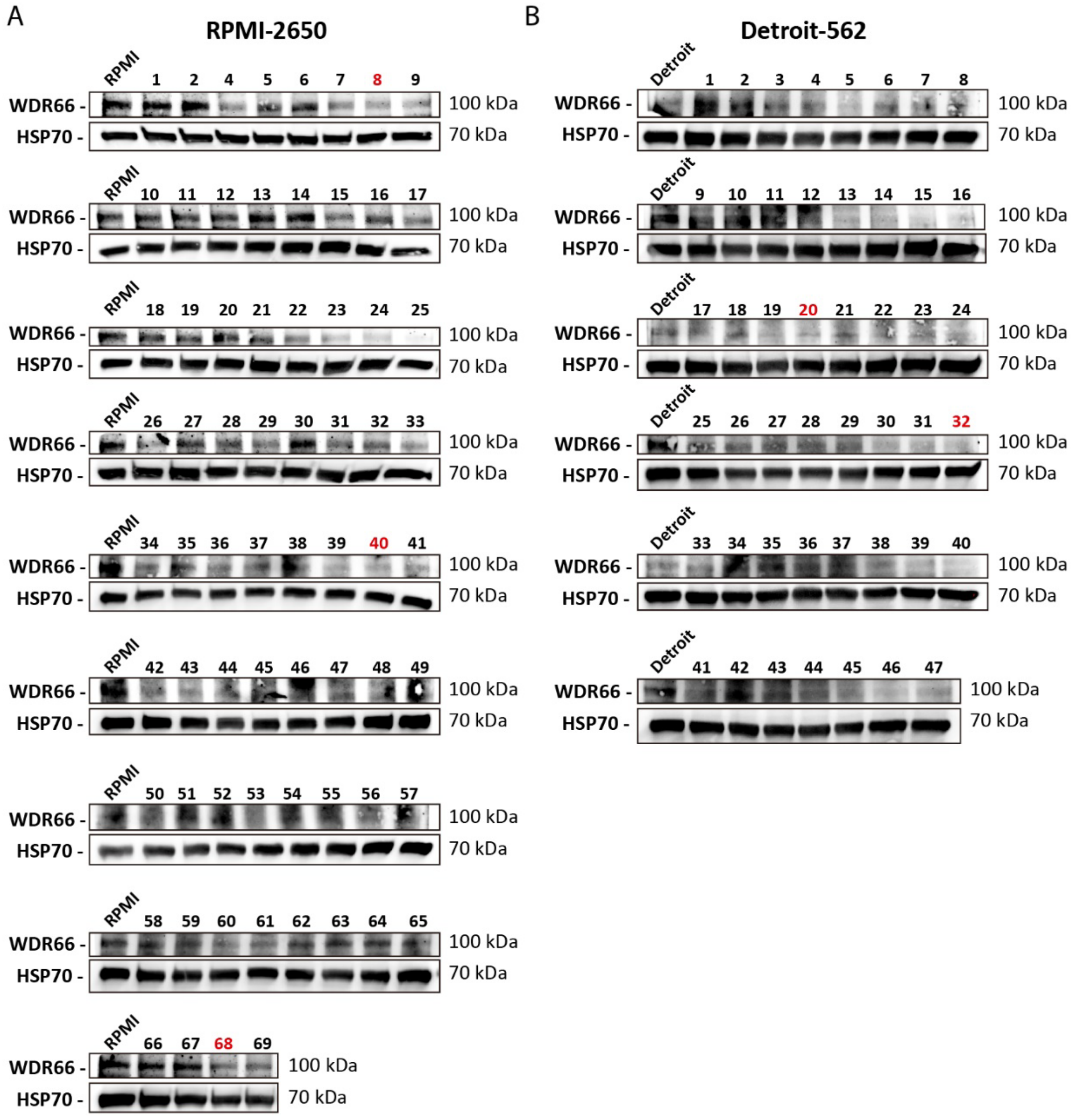

Supplementary figure 2. *Verification of WDR66 protein levels in the generated CRISPR clones. Western blot analysis of WDR66 protein levels in CRISPR clones generated in the RPMI-2650* ***(A)*** *and Detroit-562* ***(B)*** *lines. Clones in red are the ones ultimately selected for this study.*

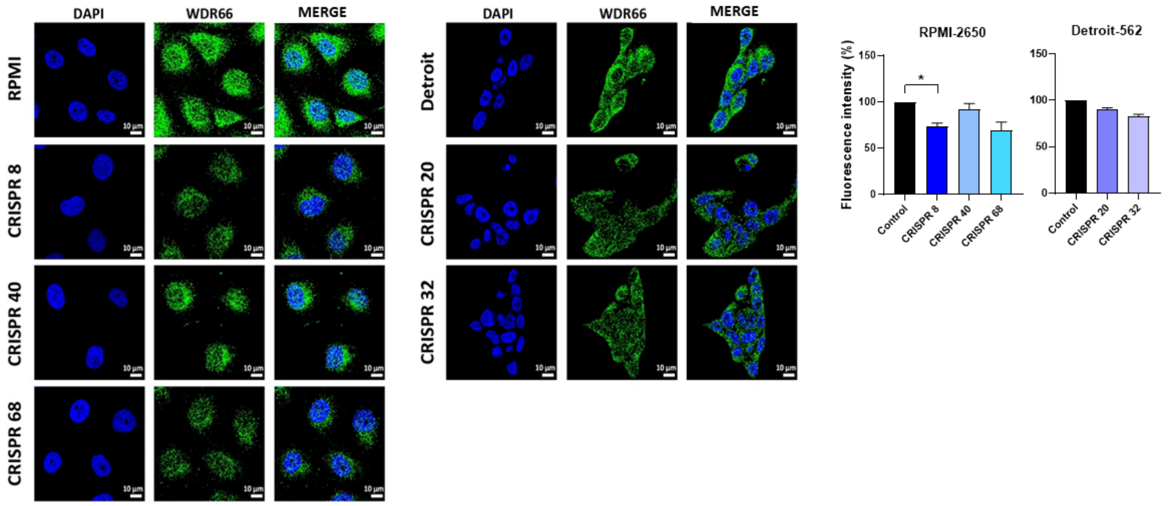

Supplementary Figure 3. *Verification of the expression level of WDR66 in CRISPR clones. Study of the WDR66 protein level by immunofluorescence in CRISPR and parental lines RPMI-2650 and Detroit-562. Representative images of WDR66 labeling and DAPI staining are shown. Median fluorescence intensity was quantified using ImageJ in at least 10 images per condition from three independent biological replicates (>100 cells per condition). Data are shown as mean ± SEM. Statistical significance was determined using an unpaired Student's t-test with Welch's correction.*

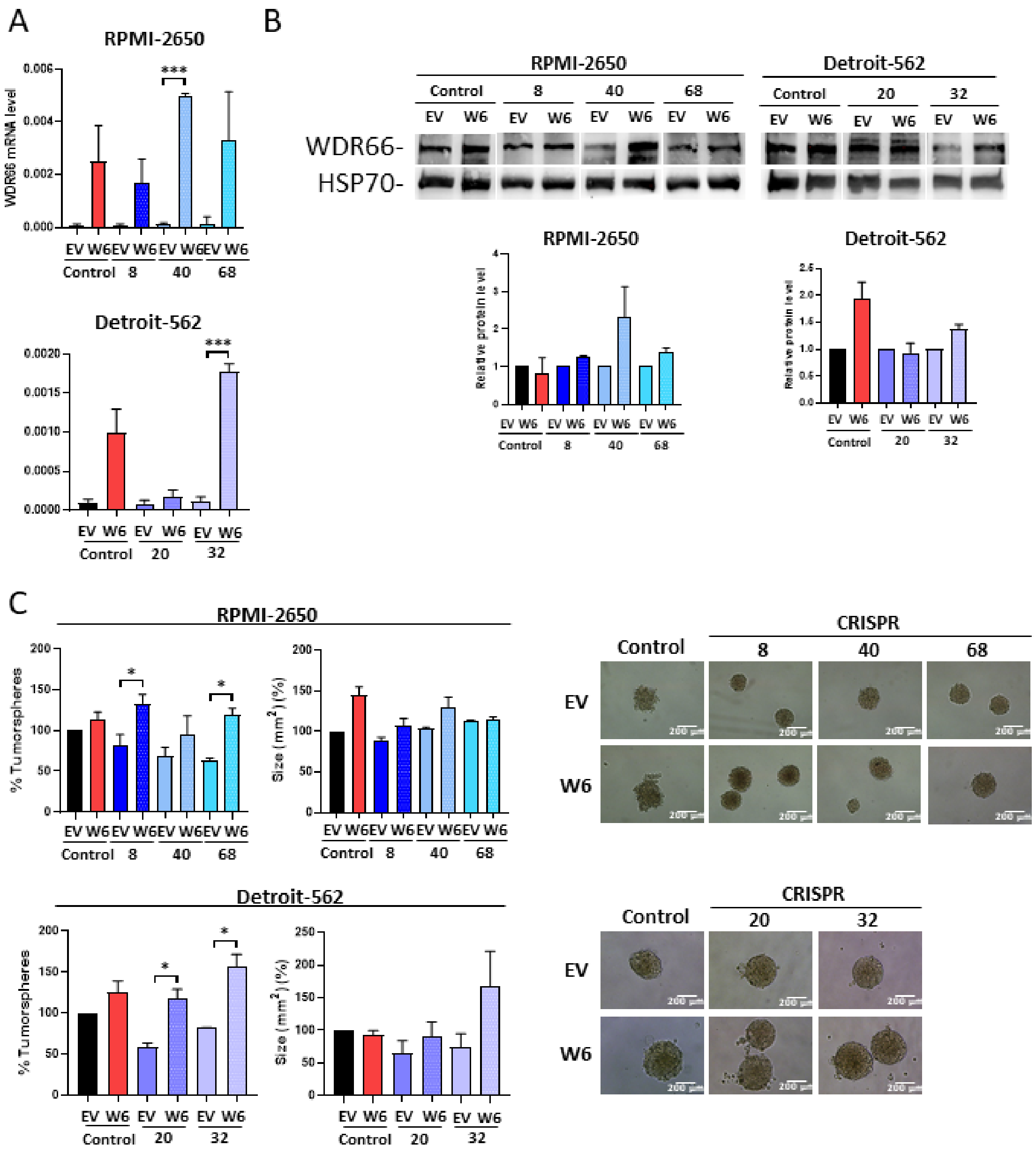

***Supplementary Figure 4. Rescue of WDR66 expression in CRISPR clones and its effect on stemness-associated properties in head and neck cancer cell lines. (A-B)** Validation of mRNA and protein levels in CRISPR clones by **(A)** RT–qPCR analysis and **(B)** Western blot analysis. **(C)** Tumorsphere formation assay, including number and size of tumorspheres. The mean of 3 independent experiments ± SEM is presented. Statistical analysis was performed with Student's t test (*p<0.05; **p<0.01; ***p<0.001). The absence of an asterisk means that the data are not statistically significant.*

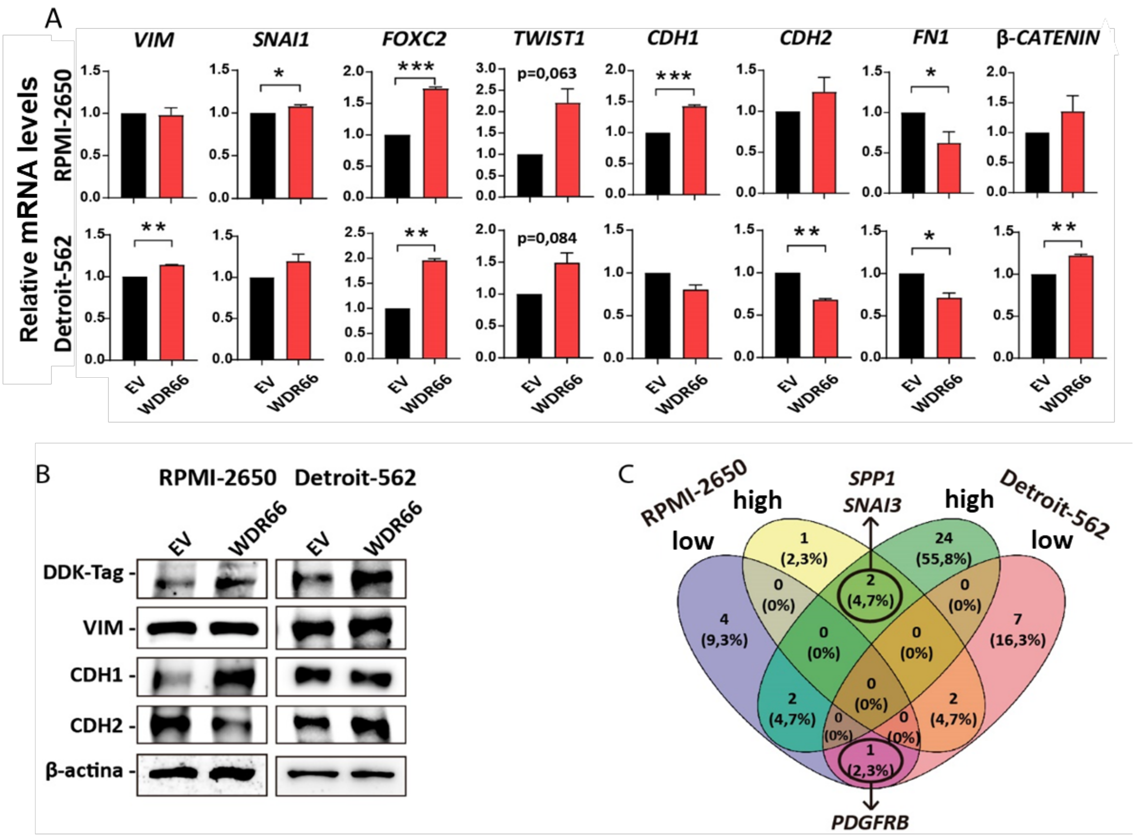

***Supplementary figure 5**. Effect of WDR66 overexpression on epithelial–mesenchymal transition (EMT) in head and neck cancer cell lines. **(A)** Study of EMT-related gene expression by RT-qPCR in control and WDR66-overexpressing cells from RPMI-2650 and Detroit-562 lines. **(B)** Study of protein levels of EMT-related genes by Western blot in control and WDR66-overexpressing cells from RPMI-2650 and Detroit-562 lines. **(C)** PCR-array of EMT-related gene expression in WDR66-overexpressing cells and control cells from RPMI-2650 and Detroit-562. The assays represent the mean of 3 independent experiments performed in triplicate ± standard error. Statistical analysis was performed using the Student t-test: *p<0.05, **p<0.01, ***p<0.001; if no asterisk is shown, the statistic was not significant. EV=Empty vector; WDR66= WDR66 overexpressing cells.*

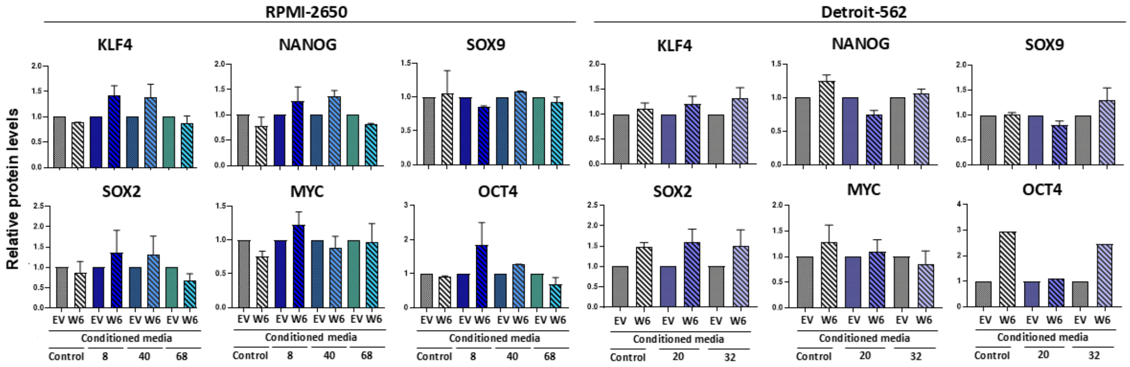

***Supplementary Figure 6. Quantification of stemness-related proteins in conditioned medium from EV and WDR66 conditions in both cell lines.** Protein levels were normalized to β-actin and expressed relative to their respective control conditions (set to 1). Data are shown as mean ± SEM. Statistical significance was determined using Student's t-test with Welch's correction.*

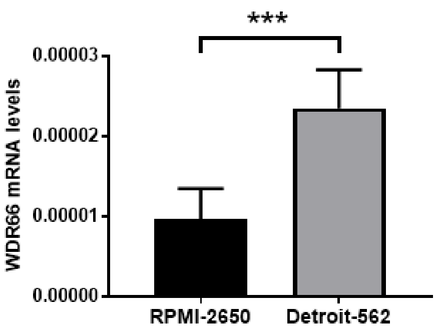

Supplementary Fig 7. mRNA expression levels of WDR66 in HNSCC cell lines. *Relative expression of WDR66 measured by RT–qPCR in the two head and neck cancer cell line, RPMI-2650 and Detroit-562. Data are presented as mean ± SEM of at least three independent experiments. Statistical analysis was performed using Student's t test (*p<0.05; **p<0.01; ***p<0.001).*
